# Diurnal changes in pathogenic and indicator virus concentrations in wastewater

**DOI:** 10.1007/s11356-023-30381-3

**Published:** 2023-11-22

**Authors:** Kata Farkas, Igor Pântea, Nick Woodhall, Denis Williams, Kathryn Lambert-Slosarska, Rachel C. Williams, Jasmine M. S. Grimsley, Andrew C. Singer, Davey L. Jones

**Affiliations:** 1 School of Environmental Natural Sciences, Bangor University, Bangor, LL57 2UW Gwynedd UK; 2 Data Analytics & Surveillance Division, UK Health Security Agency, 10 South Colonnade, Canary Wharf, London, E14 4PU UK; 3 The London Data Company, London, EC2N 2AT UK; 4 UK Centre for Ecology & Hydrology, Wallingford, OX10 8BB UK; 5 Food Futures Institute, Murdoch University, Murdoch, WA 6150 Australia

**Keywords:** Environmental monitoring, Human viruses, Indicator virus, Public health, RT-qPCR, Sewage surveillance

## Abstract

Wastewater-based epidemiology (WBE) has been commonly used for monitoring SARS-CoV-2 outbreaks. As sampling times and methods (i.e. grab vs composite) may vary, diurnal changes of viral concentrations in sewage should be better understood. In this study, we collected untreated wastewater samples hourly for 4 days at two wastewater treatment plants in Wales to establish diurnal patterns in virus concentrations and the physico-chemical properties of the water. Simultaneously, we also trialled three absorbent materials as passive samples as a simple and cost-efficient alternative for the collection of composite samples. Ninety-six percent of all liquid samples (*n* = 74) and 88% of the passive samplers (*n* = 59) were positive for SARS-CoV-2, whereas 87% and 97% of the liquid and passive samples were positive for the faecal indicator virus crAssphage, respectively. We found no significant daily variations in the concentration of the target viruses, ammonium and orthophosphate, and the pH and electrical conductivity levels were also stable. Weak positive correlations were found between some physico-chemical properties and viral concentrations. More variation was observed in samples taken from the influent stream as opposed to those taken from the influent tank. Of the absorbent materials trialled as passive samples, we found that tampons provided higher viral recoveries than electronegative filter paper and cotton gauze swabs. For all materials tested, viral recovery was dependent on the virus type. Our results indicate that grab samples may provide representative alternatives to 24-h composite samples if taken from the influent tank, hence reducing the costs of sampling for WBE programmes. Tampons are also viable alternatives for cost-efficient sampling; however, viral recovery should be optimised prior to use.

**Supplementary Information:**

The online version contains supplementary material available at 10.1007/s11356-023-30381-3.

## Introduction

SARS-CoV-2 is a novel coronavirus that was first detected in Wuhan, China, in December 2019. As of 25 August 2023, the spread of this virus has led to the COVID-19 pandemic; there have been 770 million registered cases and 7 million deaths associated with COVID-19 worldwide (WHO 2020). SARS-CoV-2 is a respiratory pathogen with effects on individuals ranging from asymptomatic carriage to mild and severe symptoms which may ultimately result in death (Zhang et al. 2022). As clinical surveillance tends to be biased towards symptomatic cases, it may underestimate true case numbers of COVID-19 within a population (Zhao et al. 2020). Despite being a respiratory pathogen, SARS-CoV-2 has been detected in the faeces of both symptomatic and asymptomatic individuals (Zhang et al. 2021). Therefore, routine monitoring of the virus in sewage has been implemented in many countries to capture the prevalence rates and describe circulating variants of SARS-CoV-2 within urban communities (Hill et al. 2021; Fuschi et al. 2021; Pillay et al. 2021; Brunner et al. 2022).

Human-derived wastewater has been used previously for tracking the use of a wide range of chemicals (e.g. pharmaceuticals, illicit drugs, antibiotics) and public health markers such as enteric viruses (González-Mariño et al. 2020; Ahmed et al. 2020a; Chacón et al. 2021; Elder et al. 2021; Huizer et al. 2021). This has led to the development of wastewater-based epidemiology (WBE) as a rapidly emerging field (Levy et al. 2023). By detecting and quantifying levels of SARS-CoV-2 in wastewater, temporal changes in viral concentrations can be tracked and used as a complimentary monitoring tool alongside confirmed clinical case numbers (Wade et al. 2022). Viral concentrations can be monitored at a community level on large scales by taking samples from wastewater treatment plants (WWTPs), or on a local scale by taking samples near to source, for example, at hospitals, airports, prisons and university campuses (Kapoor et al. 2022; Jain et al. 2022). WBE may act as an early warning system for potential new outbreaks and re-emergence of the virus, with increases in viral concentrations in wastewater preceding increases detected by clinical cases (Peccia et al. 2020; Aguiar-Oliveira et al. 2020). The wastewater viral concentration changes can be used to advise on and implement local or national policies on lockdowns, vaccination drives and awareness campaigns (Wurtzer et al. 2020; Medema et al. 2020).

While WBE has become an important tool in outbreak surveillance, it is not without its limitations. For example, viral concentrations in wastewater may be affected by dilution from non-human sources (e.g. by rainfall), by diurnal patterns in bathroom use, pumping within the sewer network, or due to variation in viral quantification methods used for testing (Ahmed et al. 2020b; Farkas et al. 2022). Furthermore, data normalisation for populations may also be challenging due to the lack of supporting data (Wilder et al. 2021). A robust sampling strategy is crucial in WBE to enable accurate sample analysis. Due to human behaviour and environmental conditions, the viral load in wastewater varies over time. For instance, Birks and Hills (2007) found peak flows of wastewater at treatment plants which tend to occur around 08:00 h and 22:00 h, with lulls around 05:00 h and 15:00 h. The highest concentrations of human-derived compounds (faecal indicator bacteria, hormones, antibiotics) have been shown to occur at times of the highest flows (Plósz et al. 2010; Ekklesia et al. 2015), suggesting that the timing of sampling is an important consideration in WBE (Gerba et al. 2017). While some studies have suggested that human virus (e.g. SARS-CoV-2, enteroviruses, noroviruses, sapoviruses) and faecal indicator virus (e.g. human adenoviruses, pepper mild mottle virus, coliphages) concentrations vary during the day (Ahmed et al. 2021; Bivins et al. 2021), other studies have found no distinct diurnal peaks in virus concentrations in wastewater (Kim et al. 2009; Farkas et al. 2018a). More studies conducting high-frequency sampling are therefore necessary to investigate viral diurnal variations of human-derived viruses in wastewater.

Wastewater surveillance typically consists of taking one sample a day which could either be a grab or composite sample. Grab samples can be taken by hand or machine if available and are a low cost, reliable option. However, given the diurnal variation, there is the potential for this method to miss peak viral loads in the sewage network, therefore underestimating viral concentrations (Augusto et al. 2022). Furthermore, there is a potential for less consistency between daily samples when the sampling time or peak flow varies between days. A composite sample taken over 24 h captures small volumes of sample throughout the day, eliminating single sample time points. While more likely to capture novel viruses more consistently between days, actual concentration/quantification estimates may be lower than a grab sample taken at peak load time due to dilution in the sample collection bottle (Gerba et al. 2017). Composite samples are best taken with an autosampler, which may be expensive or hard to deploy at sampling sites (Bivins et al. 2022a). Furthermore, it is also possible that the genetic material may degrade in wastewater over longer time periods (e.g. in autosampler bottles), especially if they are not refrigerated (McCall et al. 2022), introducing a potential for weather-dependent impacts on viral levels.

To overcome the limitations of using an autosampler, passive samplers may be deployed for capturing viruses in wastewater. These can be constructed at low cost using commercially available absorbent materials, such as cheesecloth, tampons, cotton gauze, cotton buds and filter papers (Bivins et al. 2022b; Hayes et al. 2021b, 2021a; Kevill et al. 2022a; Liu et al. 2022; Schang et al. 2021). Generally, the sampling material is housed in an outer casing, a “torpedo” or “boat”, to prevent fouling and ragging while exposed to wastewater (Wilson et al. 2022). They can be advantageous in situations where the water flow is highly variable or in deep sewers where autosamplers may fail to work effectively. Passive samplers also allow for near source monitoring which is often not possible for autosamplers due to their size (Liu et al. 2022).

In this study, we used autosamplers for hourly wastewater sampling to investigate short-term diurnal changes of viral load in wastewater influent at two urban wastewater treatment plants, focusing on SARS-CoV-2 and the faecal indicator virus, crAssphage. We selected these viruses due to their high abundance in wastewater at the sampling sites during the time of sampling. In addition, we evaluated the potential benefits of using passive samplers, testing three different materials for their durability and viral saturation point, directly alongside the autosamplers.

## Materials and methods

### Sampling sites and procedures

Untreated wastewater influent samples were collected from two WWTPs located in the UK, Chester and Kinmel Bay, serving 105,571 and 48,234 inhabitants, with mean flows of 252 and 149 l s^−1^, respectively. Samples were collected between 2 and 6 August 2021 at the direct inlet stream behind the primary screen at Chester WWTP (53°11′30″N 2°54′38″W) and from the influent tank at Kinmel Bay WWTP (53°18′38″N 3°31′11.6″W) between 9 and 13 August 2021. Only one rain event was observed at Chester (6 August 2021, 4–9 am; 8.8 mm) during the sampling periods (CEDA Archive, https://data.ceda.ac.uk/).

Wastewater samples were taken hourly using two Avalanche-refrigerated autosamplers (Teledyne ISCO, Lincoln, NE, USA). Typically, we collected composite samples with a total volume of 0.9 l; however, occasionally smaller volumes were collected due to pipe clogging. The samples were collected every day for 4 days and brought to the laboratory chilled (4 °C) for further processing and analysis. Overall, 83 and 91 samples were collected at Chester and Kinmel Bay, respectively.

Along with the autosamplers, passive samplers were also deployed and collected daily. We trialled three sampler materials, namely Tampax Compak Super tampons (Procter & Gamble UK), SG81 silica-cellulose electronegative filter paper (Whatman, UK), and cotton gauze (Moore) swabs. Further details of the chemical and physical properties of the passive samplers have been discussed previously (Jones et al. 2022). Triplicates of each passive sampler material were placed in polypropylene mesh cages in the wastewater stream. The samples were recovered after 24 h and transported back to the laboratory chilled for further sample processing and analysis. We deployed, when possible, triplicates of each sampler type once a day at Chester (*n* = 12 for each sampler type) and in duplicates twice a day at Kinmel Bay (*n* = 12 for each sampler type). However, only 11 Tampax, 5 paper and 7 cotton samplers were recovered at Kinmel Bay due to high water flow.

### Physico-chemical analyses

Wastewater electrical conductivity (EC) was measured using a Jenway 4520 conductivity meter and pH with a Hanna 209 pH meter (Hanna Instruments Ltd., Leighton Buzzard, UK). Wastewater ammonium concentrations were determined colorimetrically using the salicylic acid procedure of Mulvaney (1996). Molybdate-reactive orthophosphate was determined colorimetrically according to the molybdate blue procedure of Murphy and Riley (1962).

### Sample process for viral detection

For virus detection, the liquid wastewater samples were concentrated using polyethylene glycol (PEG) precipitation as described in Farkas et al. (2021). With each set of samples, a control with 18 MΩ resistance deionised water was also processed. In brief, 200 ml of each sample was centrifuged to eliminate solid matter and then 150 ml of the supernatant mixed with PEG8000 and NaCl to reach a final concentration of 10% and 2%, respectively. Following a 16-h incubation at 4 °C, the samples were centrifuged, and the viral nucleic acids were extracted directly from the pellet using the NucliSense extraction system (BioMerieux, France) on the KingFisher 96 Flex system (Thermo Scientific, USA) as described elsewhere (Farkas et al. 2021; Kevill et al. 2022b). A 0.2 ml aliquot of the 150 ml supernatant was also subject to nucleic acid extraction. On each extraction plate, 2–4 extraction negatives, consisting of 0.2 ml phosphate saline buffer (PBS) pH 7.4, were included. The final volume of the extracts was 0.1 ml.

A 1-cm^2^ piece of the passive sampler material was subject to direct nucleic acid extraction as described previously (Kevill et al. 2022a). For extraction control, 0.5 ml PBS was used. The samples and controls were mixed with 2 ml of NucliSens lysis buffer (BioMerieux, France), vortexed for 10 s and incubated at room temperature for 10 min. Subsequently, the sampling material was squeezed to elute all the remaining liquid and removed. The samplers were then extracted using the MiniMag NucliSens extraction reagents (BioMerieux, France) as described elsewhere (Farkas et al. 2021).

### Quantification of viral nucleic acids

The (RT-)qPCR assays were performed on a QuantStudio® Flex 6 Real-Time PCR System (Applied Biosystems, USA). SARS-CoV-2 RNA was detected using the N1 primer–probe set (CDC 2020). For crAssphage, we used an established primer–probe set (Stachler et al. 2017). SARS-CoV-2 was quantified using the TaqMan 1-step Virus RT-qPCR kit (Invitrogen, USA) with synthetic RNA standards, as described elsewhere (Farkas et al. 2022). CrAssphage was quantified using the QuantiFast probe PCR mix (Qiagen, Germany) with plasmid DNA standards, as described elsewhere (Kevill et al. 2022b). Each reaction plate contained four non-template controls, which were negative for all targets.

### Data analysis

Viral concentrations were expressed as genome copies (gc) in 1 l wastewater or in 1 cm^2^ of passive sampler material. Concentration efficiency was calculated by dividing the crAssphage concentration in concentrated wastewater by the crAssphage concentration in raw samples and expressed in percentiles. To compare the efficiency of passive samplers and liquid wastewater samples for virus recovery, relative concentrations were calculated by dividing passive sampler virus concentrations (gc/cm^2^) by liquid wastewater virus concentrations (gc/l).

The “rcorr” function in R v4.1.2 (R Core Team 2021) was used to compute Spearman’s rank correlations for all wastewater parameters except for sampling time. The results were plotted with “corrplot”. In order to investigate the differences of the wastewater parameters at different time intervals, the data was divided in 12-, 8-, 6-, 4-, 3-h interval groups, plotted as boxplots (i.e. minimum, first quartile, median, third quartile and maximum) and compared against each other with the non-parametric Wilcoxon rank-sum test in R. For visualisation, additional graphs were produced separately in Python v3.10.0 (Python Software Foundation, 2022) using the “matplotlib.pyplot” library with a polynomial trendline of 10th order (functions “numpy.poly1d” and “numpy.polyval”) and a Gaussian trendline (“gaussian_filter1d”) (Table S1).

A Shapiro–Wilk test was applied for the passive samplers’ comparisons groups to determine whether the data follows an approximately normal distribution. Since some of the passive samplers’ groups had a Shapiro–Wilk test *p*-value < 0.05, Mann–Whitney *U* test was used to compare the performance of passive samplers in R.

## Results

### Quality control

The extraction and qPCR negative controls were negative throughout the study suggesting no cross-contamination. The qPCR standard curve slope, R^2^ and efficiency (Table 1) were all within the acceptable range. The low limit of detection (LOD) and limit of quantification (LOQ) values for the qPCR assays used (Table 1) suggested high sensitivity. The sample concentration efficiency calculated for crAssphage varied between 0.02 and 131% in the samples collected at Chester (mean: 6.43%) and between 0.54 and 35% at Kinmel Bay (mean: 4.99%).

**Table 1:** Standard curve slope, efficiency and R^2^ limit of detection (LOD) and limit of quantification (LOQ) values for each target virus using RT-qPCR and qPCR for SARS-CoV-2 and crAssphage, respectively. LOD and LOQ values were adopted from Farkas et al. (2022)

	Slope	Efficiency (%)	*R*^2^	LOD (gc/µl extract)	LOQ (gc/µl extract)
SARS-CoV-2	− 3.321 ± 0.118	100.2 ± 5.0	0.992 ± 0.008	0.9	12.6
CrAssphage	− 3.302 ± 0.105	101.0 ± 4.4	0.997 ± 0.002	2.31	12.5

### Diurnal variations in wastewater physico-chemical properties

In a 24-h period, similarity was shown between the two WWTPs in wastewater parameters such as pH, orthophosphate concentration and ammonia concentration (Table 2). Greater divergence was shown in other parameters, particularly wastewater turbidity and EC (Table 2, Figures S1-S5).

**Table 2:** The mean, standard deviation (SD), 95% confidence interval (CI), the sample size (*n*) and the standard error mean (SEM) for the collected liquid samples collected at the Chester and Kinmel Bay WWTPs

Parameter	Mean	SD	95% CI	*n*	SEM
Chester					
SARS-CoV-2 concentration (log_10_ gc/l)	4.58	4.56	4.48–4.66	76/83	3.60
CrAssphage concentration (log_10_ gc/l)	9.12	9.66	8.47–9.37	54/75	8.72
pH	7.54	0.17	7.50–7.57	81	0.02
Electrical conductivity (µS/cm)	1133	702	980–1286	81	78
Turbidity (NTU)	228	118	203–254	81	13
Ammonium concentration (mg/l)	37.9	9.8	35.7–40.0	81	1.1
Phosphate concentration (mg/l)	3.05	1.38	2.75–3.35	81	0.15
Kinmel Bay					
SARS-CoV-2 concentration (log_10_ gc/l)	4.65	4.69	4.54–4.74	91/91	3.71
CrAssphage concentration (log_10_ gc/l)	8.63	8.31	8.58–8.67	89/89	7.34
pH	7.44	0.13	7.41–7.47	77	0.01
Electrical conductivity (µS/cm)	4903	1316	4609–5197	77	150
Turbidity (NTU)	115	27	109–121	77	3
Ammonium concentration (mg/l)	33.9	3.9	33.1–34.8	77	0.4
Phosphate concentration (mg/l)	4.04	1.17	3.78–4.3	76	0.13

More than 95% of samples were within the pH range of 7.2–7.8; however, pH during daytime showed some variation with different trends at Chester and Kinmel Bay (Figures S1, S6-7). The pH increased for Chester samples between 06:00 and 10:00 h, whereas at Kinmel Bay, the increase was observed later, between 08:00 and 12:00 h. The wastewater turbidity was considerably higher at Chester compared to Kinmel Bay. At Chester, the peak in turbidity was observed at 12:00–14:00 h (Figure S2), whereas the lowest turbidity levels occurred in the early morning hours (05:00–08:00 h; Figure S8). The pH and turbidity levels in the samples from Kinmel Bay samples lacked any distinct diurnal variation (Figures S1-2, S6-9).

The ammonium and orthophosphate concentrations also varied in samples taken at Chester (Figures S3-4, S10, S12). Major peaks were observed late morning (09:00–14:00 h), shortly after the pH peak. The orthophosphate concentration varied more in the Chester samples, similar to ammonium, with an increase starting at 07:30 h, peaking at 11:00–12:00 h, followed by a gradual decrease and relative stabilisation at 16:00 h (Figures S4, S12). Although less variable, the Kinmel Bay trends for ammonium and orthophosphate are similar with a small increase in concentration between 10:00 and 13:00 h (Figures S3-4, S11, S13).

The EC of the samples collected at Kinmel Bay showed peaks in the morning (08:00 h) and evening (18:00 h; Figure S5). Significantly higher EC values were observed in the morning hours (07:00–11:00 h) and late afternoon (15:00–19:00 h) than at midday, in the evening and at night (Figure S15). In contrast, the EC values in the Chester samples centred around the mean value without distinct diurnal peaks or patterns (Figure S5, S14).

### Diurnal variations in virus concentrations in wastewater

**Fig. 1:**
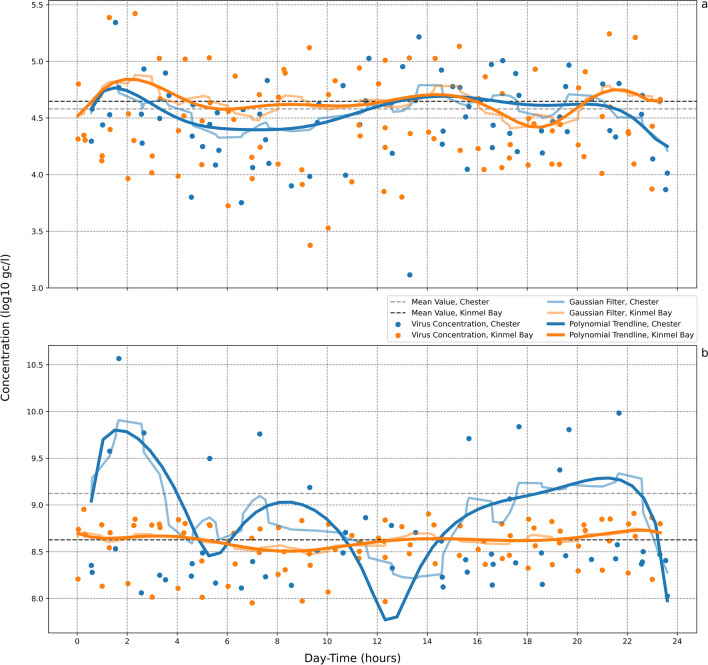
Diurnal variation of **a** SARS-CoV-2 and **b** crAssphage concentrations in wastewater samples collected at Chester (blue) and Kinmel Bay (orange) WWTPs. The polynomial function and a Gaussian function filter, sigma = 2, were applied to observe the trend during the day

At Chester, 92% of the collected samples were positive for SARS-CoV-2 with a mean concentration of 4.58 log_10_ gc/l (Table 2). A gradual increase of SARS-CoV-2 concentration in the samples was noted with approximately 4.3 log_10_ gc/l at 7:30 h, peaking at 13:30 h with a concentration of 4.7 log_10_ gc/l (Fig. 1). At Kinmel Bay, all samples were positive for SARS-CoV-2 with a mean concentration of 4.65 log_10_ gc/l (Table 2), and the samples demonstrated only slight increases in virus concentrations at 01:00 h, 14:00 h and 21:30 h (Fig. 1). No significant diurnal variations in SARS-CoV-2 concentrations were observed at either sampling site (Figures S16-17).

For crAssphage, 72% and 100% of the collected samples were positive at Chester and Kinmel Bay, respectively. A decrease in crAssphage concentration was observed in Chester at 14:30 h (Fig. 1). Although the crAssphage concentration oscillated between 8 and 9 log_10_ gc/l for the Kinmel Bay samples (Fig. 1), the polynomial trendline was relatively stable. No significant diurnal variations in crAssphage concentrations were observed at either sampling site (Figures S18-19).

### Correlation between physico-chemical properties and viral concentrations in wastewater

**Fig. 2:**
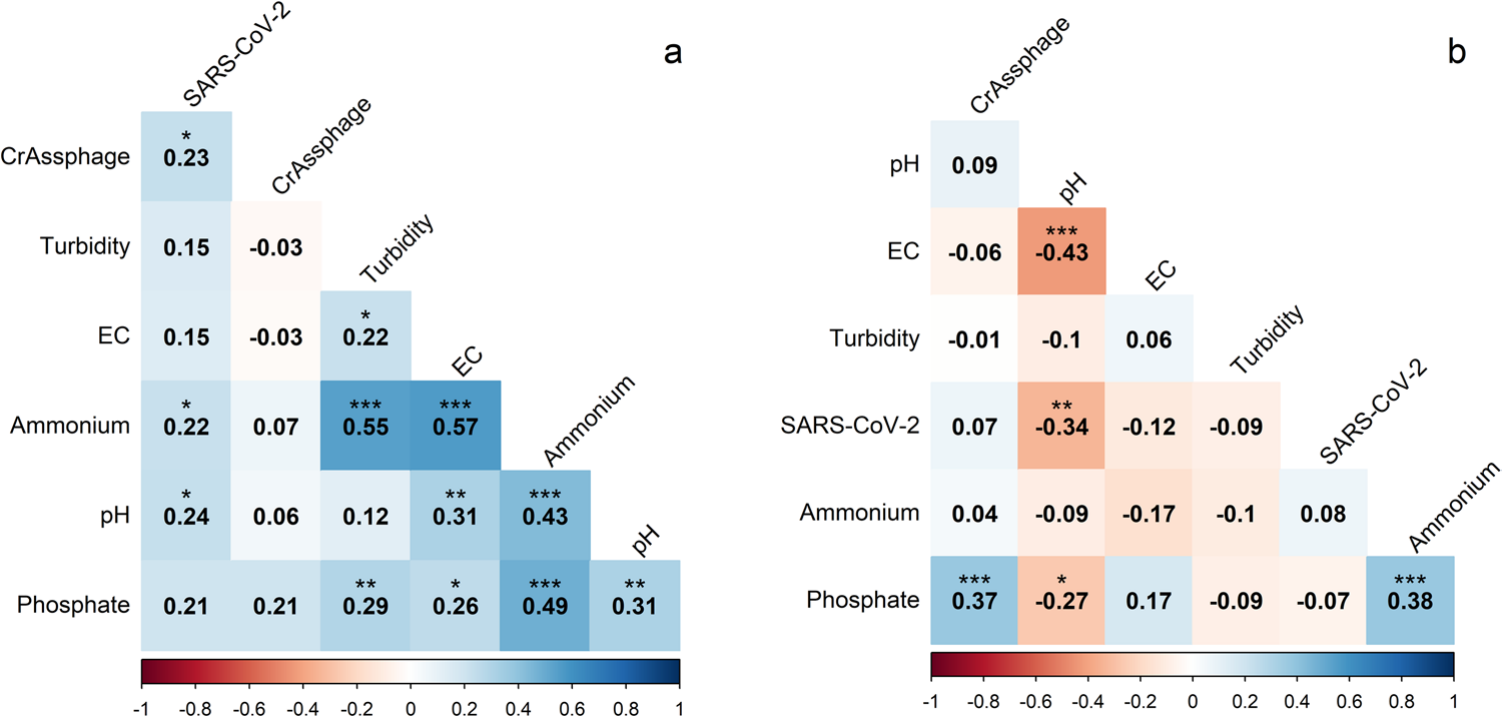
Spearman’s rank correlation coefficients established between viral concentrations and physico-chemical properties of wastewater at **a** Chester and **b** Kinmel Bay wastewater treatment plants (***p < 0.001; **p < 0.01; *p < 0.05)

At Chester, a moderate positive correlation was observed between crAssphage and SARS-CoV-2 titres using Spearman’s rank correlation (Fig. 2). A similar relationship was observed between ammonium levels and pH, phosphate, turbidity or EC levels. A weaker positive correlation was noted between the remaining tested physico-chemical properties. Interestingly, at Kinmel Bay, a negative correlation was observed between pH and SARS-CoV-2, EC or phosphate. A weak positive correlation was also observed between phosphate and crAssphage or ammonium levels (Fig. 2).

### Comparative assessment of passive samplers

**Fig. 3:**
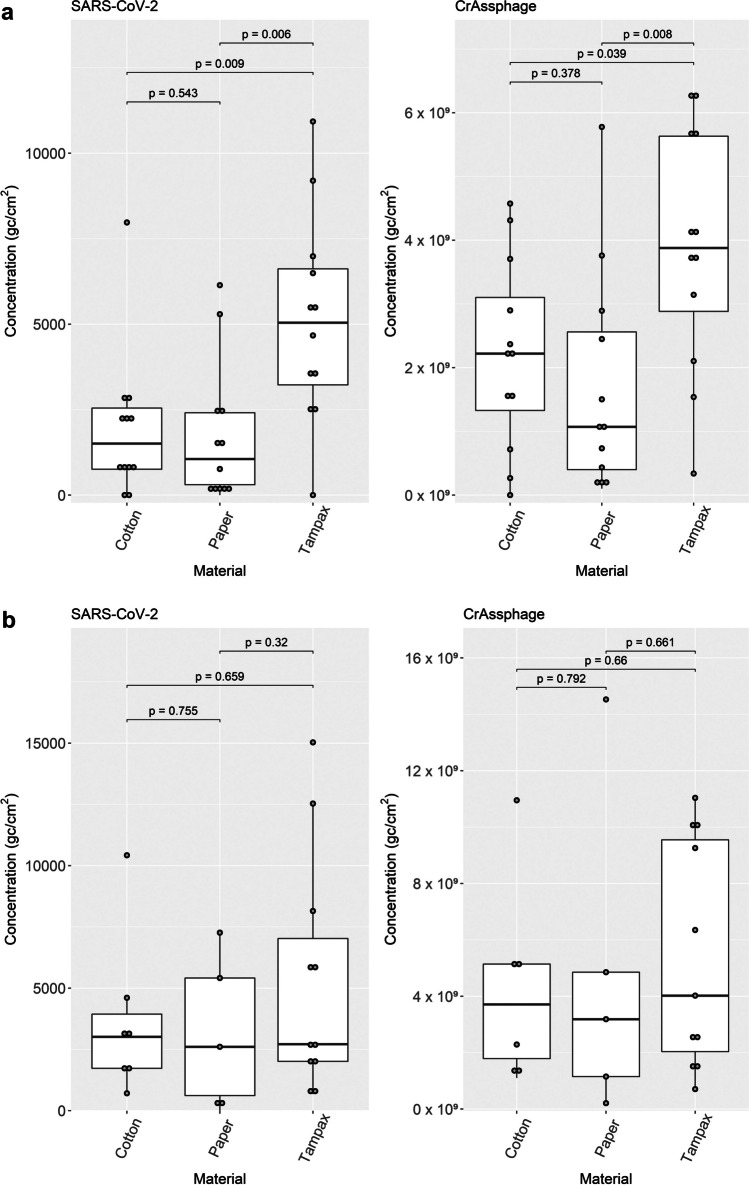
Comparison of the virus recovery by passive sampler type in samples collected at **a** Chester WWTP and **b** Kinmel Bay WWTP. SARS-CoV-2 concentrations are on the left, crAssphage concentrations are on the right. Comparisons were made with a Wilcoxon rank-sum test, the results being represented by the corresponding p-value. The boxes correspond to the interquartile range, 25th, 50th and 75th percentile range, while the middle line of the box corresponds to the median value. The whiskers correspond to the minimum and maximum value. Data points outside the whisker range represent outliers omitted from the calculation of the interquartile range

The Tampax passive sampler performed significantly better than the paper or cotton samplers for capturing both SARS-CoV-2 and crAssphage at the Chester WWTP (Fig. 3). Cotton samplers had higher median SARS-CoV-2 and crAssphage recoveries than the paper-based ones; however, the difference was not significant. At Kinmel Bay, the Tampax passive sampler had higher median SARS-CoV-2 and crAssphage concentrations, followed by cotton and then paper samplers; however, the differences were not significant (Fig. 3).

A direct comparison was not possible between the concentrations detected by liquid samples derived from autosamplers, and the material of passive samplers due to the differences in sampling and sample processing. Therefore, daily relative concentrations were calculated at each WWTP to assess viral recovery efficiency (Table 3). The relative concentrations were below 1 for SARS-CoV-2 and mostly above 1 for crAssphage at both sites for all three types of passive samplers. This suggests that the passive sampler elution can recover crAssphage more efficiently than the PEG precipitation method applied for liquid samples, whereas the opposite trends are observed for SARS-CoV-2.

**Table 3:** Relative viral concentrations (gc/cm^2^ concentration measured in passive samplers divided by gc/l concentrations measured in liquid wastewater) calculated for each sampling day at the Chester and Kinmel Bay WWTPs

Sampler	Date	SARS-CoV-2	CrAssphage
Chester WWTP			
Tampax	03/08/2021	0.11	15.76
	04/08/2021	0.10	2.81
	05/08/2021	0.19	1.71
	06/08/2021	0.23	1.88
Cotton	03/08/2021	0.03	5.10
	04/08/2021	0.02	1.26
	05/08/2021	0.13	0.85
	06/08/2021	0.09	1.74
Paper	03/08/2021	0.05	3.11
	04/08/2021	0.03	1.14
	05/08/2021	0.09	1.01
	06/08/2021	0.05	0.75
Kinmel Bay WWTP			
Tampax	10/08/2021	0.07	17.71
	11/08/2021	0.04	5.02
	12/08/2021	0.28	11.62
	13/08/2021	0.07	11.99
Cotton	10/08/2021	0.09	19.88
	12/08/2021	0.11	6.63
	13/08/2021	0.05	NA
Paper	12/08/2021	0.15	9.95
	13/08/2021	0.00	2.18

## Discussion

The qPCR and RT-qPCR methods applied in this study were efficient for the detection and quantification of the target viruses (Table 1). Higher virus recoveries and negligible RT-qPCR inhibition have been observed in SARS-CoV-2 compared to crAssphage using either the PEG concentration method for liquid samples, or the direct elution from passive samplers (Kevill et al. 2022b, a; Farkas et al. 2022). Similar to the previous findings, our study also suggests that the recovery efficiency depends on a combination of virus type, sampling method and virus concentration method (Table 3).

In this study, we set up a 4-day sampling at two WWTPs to assess diurnal patterns in viral concentrations and chemical compositions. As our sampling regime was restricted by laboratory availability and limited site access, we chose 4 days of continuous sampling. Previous studies suggested that 1–3 days of continuous sampling can be used to see diurnal patterns in virus titres (Ahmed et al. 2021; Bivins et al. 2021); therefore, we believe the results accurately describe such patterns. We found different diurnal patterns in wastewater physico-chemical properties and viral concentrations at the two WWTPs; Kinmel Bay samples showed less variation than the Chester samples over time. An increase was observed around midday in the ammonium, phosphate and SARS-CoV-2 concentrations at both WWTPs and to a lesser extent in crAssphage concentration at Kinmel Bay. These results likely coincide with an increase in the human activity within the served catchment, such as increased use of toilet facilities and/or increased disposal of disinfectants and other ammonium/phosphate-containing chemicals. Correlations of these rainfall events could not be established due to the lack of rain events during the sampling periods. The significant drop in the crAssphage concentration at midday can also be related to increased industrial/cleaning activity. The lack of similar trends in SARS-CoV-2 concentrations may be due to SARS-CoV-2 RNA being more resistant to such chemicals (Bivins et al. 2020; Yang et al. 2022). In that case, crAssphage should be used for population normalisation purposes with caution (Langeveld et al. 2023).

Some correlation between viral titres and chemical properties was noted at Chester, although no such correlation was observed at Kinmel Bay. Previous studies also found little or no correlation between these parameters suggesting the chemical markers cannot be used to indicate when samples for viruses should be taken (Ottoson et al. 2006; Sidhu et al. 2017; Farkas et al. 2018b).

The viral concentrations showed some fluctuation during the day especially at the Chester site, although the differences were not significant. The lack of significant diurnal variations in the concentrations of pathogenic bacteria (*Escherichia coli, Enterococcus faecalis, Staphylococcus typhi, Pseudomonas aeruginosa*, and *Klebsiella aerogenes*), crAssphage and human adenoviruses in wastewater influent has been previously corroborated (Musyoki et al. 2013; Farkas et al. 2018a; Ahmed et al. 2021), however, some variation was noted in SARS-CoV-2 concentrations (Bivins et al. 2021). The differences in virus fluctuations may be due to the different sampling points where influent wastewater was taken. Here, the autosamplers were set up to take samples from the influent stream at Chester, which may change in properties rapidly due to its dynamic flow. No access to the influent stream was available at Kinmel Bay; therefore, the samplers were set to sample from the influent tank, where the wastewater may remain for hours resulting in less variation in physico-chemical properties and virus concentrations. Significant variation in transit time will occur in the sewer network based on distance from the WWTP which will also result in diurnal signals being dampened within the sewershed.

In this study, we evaluated the usefulness of passive samplers for the detection of viruses in wastewater. Passive samplers have been deployed to capture viruses using electronegative/positive filters, cotton- and nylon-based materials in wastewater, at WWTPs (Jones et al. 2022; Li et al. 2021; Schang et al. 2021; Vincent-Hubert et al. 2022), in sewersheds (Li et al. 2021; Hayes et al. 2021a, 2022; Habtewold et al. 2022) and in near-source settings to monitor SARS-CoV-2 at university accommodation (Bivins et al. 2022b), hospital (Wilson et al. 2022) and the Olympic village during the 2022 Olympic games (Kitajima et al. 2022). We found that that the Tampax material was superior to the filter paper and cotton swabs for the capture and recovery of SARS-CoV-2 RNA and crAssphage DNA, similar to previous studies, likely due to a higher sorption capacity and a higher resistance to high-speed flows (Jones et al. 2022; Kevill et al. 2022a). However, in some cases, electronegative membranes were superior to cotton materials (Li et al. 2021; Habtewold et al. 2022), probably due to differences in saturation times. Cotton-based materials have been shown to saturate in 6–8 h after deployment in wastewater, whereas filter membranes may uptake viruses for 24–48 h (Jones et al. 2022; Li et al. 2021). Furthermore, we were able to recover twice as many Tampax than cotton and paper samplers at Kinmel Bay due to the high water flow, further verifying the durability of Tampax samplers.

Overall, all three materials captured the target viruses in wastewater; however, the recovery of crAssphage was more efficient than the recovery of SARS-CoV-2. This may be due to different properties of the viruses (e.g. direction and density of charge of the viral surface) or subsequent differences in the efficiency of virus recovery from the materials after removal from the sewer (Hayes et al. 2021a; Kevill et al. 2022a). Nonetheless, passive samplers have been shown to capture a wide range of viruses, including coronaviruses, influenza and measles viruses, adenoviruses, noroviruses, enteroviruses and faecal indicator viruses, such as crAssphage and pepper mild mottle virus (Li et al. 2021; Wilson et al. 2022; Vincent-Hubert et al. 2022; Kevill et al. 2022a; Hayes et al. 2022). They also provide a quick, cheap and easy method to install wastewater samplings; hence, they may be applied in the future for comprehensive wastewater monitoring programmes.

## Conclusions and recommendations

Little diurnal variation in physico-chemical properties and virus concentrations were observed in the wastewater samples collected from the influent tanks at two WWTPs. Slightly elevated ammonium, orthophosphate, turbidity and viral levels were observed probably due to increased defecation activity in the community. We highlight that the time of sampling is not the only contributing factor for variation and the sampling point is also important. When the sampling is conducted from an influent tank, the constant mixing reduces variations; however, when samples are taken close to the inflow point, more variability is likely to be captured. Therefore, sampling point availability should be considered when sampling method, time and pattern are determined.

Our data suggest that representative grab samples from the influent tank may be taken at any point in the day because no major differences in SARS-CoV-2 and crAssphage concentrations were observed over time. However, sampling over the 24-h period by collecting 12 2-h composite samples of untreated influent is still recommended to observe the variability of other wastewater parameters, such as turbidity, ammonium, phosphate, pH and EC. In this study, we focused on diurnal variations in viral concentrations in wastewater influent samples, and future studies should also explore seasonal patterns in viral titres.

We found that passive samplers, specifically tampons, can be useful for tracking viruses in influent wastewater. However, the deployment time should be carefully considered to avoid saturation. As complete saturation may take 6–8 h, we recommend deployment and collection early morning and late afternoon, respectively, to capture peak human activity between 08:00 h and 16:00 h.

### Supplementary Information

Below is the link to the electronic supplementary material.

Supplementary file1 (DOCX 5602 KB)

## Data Availability

Data is available upon request. Materials are not available to share.

## References

[CR1] Ahmed F, Tscharke B, O’Brien JW (2020). Can wastewater analysis be used as a tool to assess the burden of pain treatment within a population?. Environ Res.

[CR2] Ahmed W, Bivins A, Bertsch PM (2020). Surveillance of SARS-CoV-2 RNA in wastewater: methods optimization and quality control are crucial for generating reliable public health information. Curr Opin Environ Sci Health.

[CR3] Ahmed W, Bivins A, Bertsch PM (2021). Intraday variability of indicator and pathogenic viruses in 1-h and 24-h composite wastewater samples: implications for wastewater-based epidemiology. Environ Res.

[CR4] Augusto MR, Claro ICM, Siqueira AK (2022). Sampling strategies for wastewater surveillance: evaluating the variability of SARS-COV-2 RNA concentration in composite and grab samples. J Environ Chem Eng.

[CR5] Birks R, Hills S (2007). Characterisation of indicator organisms and pathogens in domestic greywater for recycling. Environ Monit Assess.

[CR6] Bivins A, Greaves J, Fischer R (2020). Persistence of SARS-CoV-2 in water and wastewater. Environ Sci Technol Lett.

[CR7] Bivins A, North D, Wu Z (2021). Within- and between-day variability of SARS-CoV-2 RNA in municipal wastewater during periods of varying COVID-19 prevalence and positivity. ACS ES&T Water.

[CR8] Bivins A, Kaya D, Ahmed W (2022). Passive sampling to scale wastewater surveillance of infectious disease: lessons learned from COVID-19. Sci Total Environ.

[CR9] Bivins A, Lott M, Shaffer M (2022). Building-level wastewater surveillance using tampon swabs and RT-LAMP for rapid SARS-CoV-2 RNA detection. Environ Sci (camb).

[CR10] Brunner FS, Brown MR, Bassano I (2022). City-wide wastewater genomic surveillance through the successive emergence of SARS-CoV-2 Alpha and Delta variants. Water Res.

[CR11] CDC (2020). 2019-Novel Coronavirus (2019-nCoV) Real-time rRT-PCR panel primers and probes.

[CR12] Chacón L, Morales E, Valiente C (2021). Wastewater-based epidemiology of enteric viruses and surveillance of acute gastrointestinal illness outbreaks in a resource-limited region. Am J Trop Med Hyg.

[CR13] de Lourdes Aguiar-Oliveira M, Campos A, Matos AR (2020). Wastewater-based epidemiology (WBE) and viral detection in polluted surface water: a valuable tool for COVID-19 surveillance—a brief review. Int J Environ Res Public Health.

[CR14] Ekklesia E, Shanahan P, Chua LHC, Eikaas HS (2015). Temporal variation of faecal indicator bacteria in tropical urban storm drains. Water Res.

[CR15] Elder FCT, Proctor K, Barden R (2021). Spatiotemporal profiling of antibiotics and resistance genes in a river catchment: human population as the main driver of antibiotic and antibiotic resistance gene presence in the environment. Water Res.

[CR16] Farkas K, Cooper DM, McDonald JE (2018). Seasonal and spatial dynamics of enteric viruses in wastewater and in riverine and estuarine receiving waters. Sci Total Environ.

[CR17] Farkas K, Marshall M, Cooper D (2018). Seasonal and diurnal surveillance of treated and untreated wastewater for human enteric viruses. Environ Sci Pollut Res.

[CR18] Farkas K, Hillary LS, Thorpe J (2021). Concentration and quantification of SARS-CoV-2 RNA in wastewater using polyethylene glycol-based concentration and qRT-PCR. Methods Protoc.

[CR19] Farkas K, Pellett C, Alex-Sanders N et al (2022) comparative assessment of filtration- and precipitation-based methods for the concentration of SARS-CoV-2 and other viruses from wastewater. Microbiol Spectr 10. 10.1128/SPECTRUM.01102-22 10.1128/spectrum.01102-22 PMC9430619 35950856

[CR20] Fuschi C, Pu H, Negri M (2021). Wastewater-based epidemiology for managing the COVID-19 pandemic. ACS ES&T Water.

[CR21] Gerba CP, Betancourt WQ, Kitajima M (2017). How much reduction of virus is needed for recycled water: a continuous changing need for assessment?. Water Res.

[CR22] González-Mariño I, Baz-Lomba JA, Alygizakis NA (2020). Spatio-temporal assessment of illicit drug use at large scale: evidence from 7 years of international wastewater monitoring. Addiction.

[CR23] Habtewold J, McCarthy D, McBean E et al (2022) Passive sampling, a practical method for wastewater-based surveillance of SARS-CoV-2. Environ Res 204:112058. 10.1016/j.envres.2021.112058 10.1016/j.envres.2021.112058 PMC8433097 34516976

[CR24] Hayes EK, Sweeney CL, Anderson LE (2021). A novel passive sampling approach for SARS-CoV-2 in wastewater in a Canadian province with low prevalence of COVID-19. Environ Sci (camb).

[CR25] Hayes EK, Sweeney CL, Fuller M (2021). Operational constraints of detecting SARS-CoV-2 on passive samplers using electronegative filters: a kinetic and equilibrium analysis. ACS Environ Sci Technol Water.

[CR26] Hayes EK, Stoddart AK, Gagnon GA (2022) Adsorption of SARS-CoV-2 onto granular activated carbon (GAC) in wastewater: implications for improvements in passive sampling. Sci Total Environ 847. 10.1016/j.scitotenv.2022.157548 10.1016/j.scitotenv.2022.157548 PMC9308143 35882338

[CR27] Hill K, Zamyadi A, Deere D (2021). SARS-CoV-2 known and unknowns, implications for the water sector and wastewater-based epidemiology to support national responses worldwide: early review of global experiences with the COVID-19 pandemic. Water Qual Res J.

[CR28] Huizer M, ter Laak TL, de Voogt P, van Wezel AP (2021). Wastewater-based epidemiology for illicit drugs: a critical review on global data. Water Res.

[CR29] Jain N, Hamilton D, Mital S et al (2022) Long-term passive wastewater surveillance of SARS-CoV-2 for seven university dormitories in comparison to municipal surveillance. Sci Total Environ 158421. 10.1016/j.scitotenv.2022.158421 10.1016/j.scitotenv.2022.158421 PMC9433341 36058330

[CR30] Jones DL, Grimsley JMS, Kevill JL (2022). Critical evaluation of different passive sampler materials and approaches for the recovery of SARS-CoV-2, faecal-indicator viruses and bacteria from wastewater. Water (basel).

[CR31] Kapoor V, Al-Duroobi H, Phan DC et al (2022) Wastewater surveillance for SARS-CoV-2 to support return to campus: methodological considerations and data interpretation. Curr Opin Environ Sci Health 27 10.1016/j.coesh.2022.100362 PMC8975751 35402756

[CR32] Kevill JL, Pellett C, Farkas K (2022). A comparison of precipitation and filtration-based SARS-CoV-2 recovery methods and the influence of temperature, turbidity, and surfactant load in urban wastewater. Sci Total Environ.

[CR33] Kevill JL, Lambert-Slosarska K, Pellett C et al (2022a) Assessment of two types of passive sampler for the efficient recovery of SARS-CoV-2 and other viruses from wastewater. Sci Total Environ 838. 10.1016/j.scitotenv.2022.156580 10.1016/j.scitotenv.2022.156580 PMC9181630 35690190

[CR34] Kim WJ, Managaki S, Furumai H, Nakajima F (2009). Diurnal fluctuation of indicator microorganisms and intestinal viruses in combined sewer system. Water Sci Technol.

[CR35] Kitajima M, Murakami M, Iwamoto R et al (2022) COVID-19 wastewater surveillance implemented in the Tokyo 2020 Olympic and Paralympic Village. J Travel Med 29. 10.1093/JTM/TAAC004 10.1093/jtm/taac004 PMC9156005 35134222

[CR36] Langeveld J, Schilperoort R, Heijnen L (2023). Normalisation of SARS-CoV-2 concentrations in wastewater: the use of flow, electrical conductivity and crAssphage. Sci Total Environ.

[CR37] Levy JI, Andersen KG, Knight R (2023). Karthikeyan S Wastewater surveillance for public health. Science (1979).

[CR38] Li J, Verhagen R, Ahmed W (2021). In situ calibration of passive samplers for viruses in wastewater. ACS Environ Sci Technol Water.

[CR39] Liu P, Ibaraki M, VanTassell J et al (2022) A sensitive, simple, and low-cost method for COVID-19 wastewater surveillance at an institutional level. Sci Total Environ 807. 10.1016/J.SCITOTENV.2021.151047 10.1016/j.scitotenv.2021.151047 PMC8522675 34673061

[CR40] McCall C, Fang ZN, Li D (2022). Modeling SARS-CoV-2 RNA degradation in small and large sewersheds. Environ Sci (camb).

[CR41] Medema G, Been F, Heijnen L, Petterson S (2020). Implementation of environmental surveillance for SARS-CoV-2 virus to support public health decisions: opportunities and challenges. Curr Opin Environ Sci Health.

[CR42] Mulvaney RL (1996) Nitrogen - Inorganic Forms, in: Methods of Soil Analysis, Part 3. Chemical Methods (D.L. Sparks Ed.). SSSA, Madison, WI, USA, pp 1123–1184

[CR43] Murphy J, Riley JP (1962). A modified single solution method for the determination of phosphate in natural waters. Anal Chim Acta.

[CR44] Musyoki AM, Suleiman MA, Mbithi JN, Maingi JM (2013). Diurnal and seasonal variations of pathogenic bacteria in Dandora Sewage Treatment Plant wastewater, Nairobi, Kenya. J Res Environ Sci Toxicol.

[CR45] Ottoson J, Hansen A, Björlenius B (2006). Removal of viruses, parasitic protozoa and microbial indicators in conventional and membrane processes in a wastewater pilot plant. Water Res.

[CR46] Peccia J, Zulli A, Brackney DE (2020). Measurement of SARS-CoV-2 RNA in wastewater tracks community infection dynamics. Nat Biotechnol.

[CR47] Pillay L, Amoah ID, Deepnarain N (2021). Monitoring changes in COVID-19 infection using wastewater-based epidemiology: a South African perspective. Sci Total Environ.

[CR48] Plósz BG, Leknes H, Liltved H, Thomas K, v.  (2010). Diurnal variations in the occurrence and the fate of hormones and antibiotics in activated sludge wastewater treatment in Oslo, Norway. Sci Total Environ.

[CR49] R Core Team (2021) R: a language and environment for statistical computing. R Foundation for Statistical Computing, Vienna, Austria. https://www.R-project.org/

[CR50] Schang C, Crosbie ND, Nolan M (2021). Passive sampling of SARS-CoV-2 for wastewater surveillance. Environ Sci Technol.

[CR51] Sidhu JPS, Sena K, Hodgers L (2017). Comparative enteric viruses and coliphage removal during wastewater treatment processes in a sub-tropical environment. Sci Total Environ.

[CR52] Stachler E, Kelty C, Sivaganesan M (2017). Quantitative crAssphage PCR assays for human fecal pollution measurement. Environ Sci Technol.

[CR53] Vincent-Hubert F, Wacrenier C, Desdouits M et al (2022) Development of passive samplers for the detection of SARS-CoV-2 in sewage and seawater: application for the monitoring of sewage. Sci Total Environ 833. 10.1016/j.scitotenv.2022.155139 10.1016/j.scitotenv.2022.155139 PMC8993413 35405243

[CR54] Wade MJ, Lo Jacomo A, Armenise E (2022). Understanding and managing uncertainty and variability for wastewater monitoring beyond the pandemic: lessons learned from the United Kingdom national COVID-19 surveillance programmes. J Hazard Mater.

[CR55] WHO (2020) WHO Coronavirus (COVID-19) dashboard. https://covid19.who.int/. Accessed 22 Jun 2022

[CR56] Wilder ML, Middleton F, Larsen DA (2021). Co-quantification of crAssphage increases confidence in wastewater-based epidemiology for SARS-CoV-2 in low prevalence areas. Water Res X.

[CR57] Wilson M, Qiu Y, Yu J et al (2022) Comparison of auto sampling and passive sampling methods for SARS-CoV-2 detection in wastewater. Pathogens 11. 10.3390/pathogens11030359 10.3390/pathogens11030359 PMC8955177 35335683

[CR58] Wurtzer S, Marechal V, Mouchel JM (2020). Evaluation of lockdown effect on SARS-CoV-2 dynamics through viral genome quantification in waste water, Greater Paris, France, 5 March to 23 April 2020. Eurosurveillance.

[CR59] Yang S, Dong Q, Li S et al (2022) Persistence of SARS-CoV-2 RNA in wastewater after the end of the COVID-19 epidemics. J Hazard Mater 429. 10.1016/J.JHAZMAT.2022.128358 10.1016/j.jhazmat.2022.128358 PMC8800135 35123131

[CR60] Zhang Y, Cen M, Hu M (2021). Prevalence and persistent shedding of fecal SARS-CoV-2 RNA in patients with COVID-19 infection: a systematic review and meta-analysis. Clin Transl Gastroenterol.

[CR61] Zhang J, Chen N, Zhao D (2022). Clinical characteristics of COVID-19 patients infected by the Omicron variant of SARS-CoV-2. Front Med (lausanne).

[CR62] Zhao HJ, Lu XX, Deng Ybin (2020). COVID-19: asymptomatic carrier transmission is an underestimated problem. Epidemiol Infect.

