# Supplementary material for: Diurnal changes in pathogenic and indicator virus concentrations in wastewater
Source: Environ Sci Pollut Res Int. 2023 Nov 22;30(59):123785–95. doi: 10.1007/s11356-023-30381-3 (PMC10746776; doi:10.1007/s11356-023-30381-3)
Supplement: Supplementary file 1 — Supplementary file1 (DOCX 5602 KB) [file 11356_2023_30381_MOESM1_ESM.docx]

**Supplementary material for**

Diurnal changes in pathogenic and indicator virus concentrations in wastewater

Kata Farkas^a*^, Igor Pântea^a^, Nick Woodhall^a^, Denis Williams^a^, Kathryn Lambert-Slosarska^a^, Rachel C. Williams^a^, Jasmine M.S. Grimsley^b,c^ Andrew C. Singer^d^ Davey L. Jones^a,e^

^a^School of Natural Sciences, Bangor University, Bangor, Gwynedd, LL57 2UW, UK

^b^UK Health Security Agency, Data Analytics & Surveillance Division, 10 South Colonnade, Canary Wharf, London E14 4PU, UK

^c^The London Data Company, London EC2N 2AT, UK

^d^UK Centre for Ecology & Hydrology, Wallingford, OX10 8BB, UK

^e^Food Futures Institute, Murdoch University, 90 South Street, Murdoch, WA 6150, Australia

**
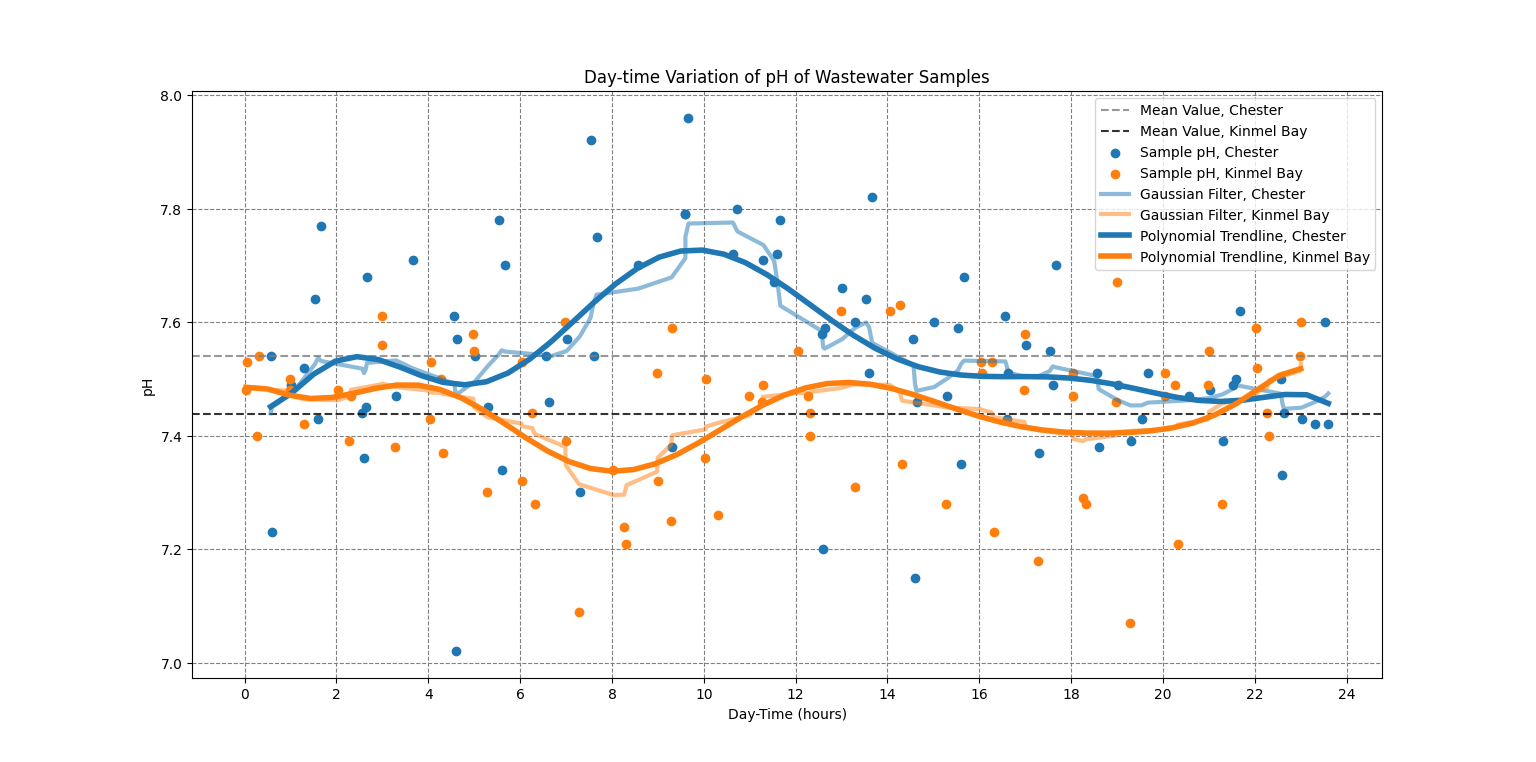
**

# **Figure S1. Diurnal Variation of Wastewater pH at the Chester and Kinmel Bay WWTPs**.

The polynomial function and a Gaussian function filter, sigma = 2, were applied to observe the trend during the day. The pH trends between Chester and Kinmel Bay mostly do not agree; however, values for both are within the narrow range of 7.2 – 7.8.


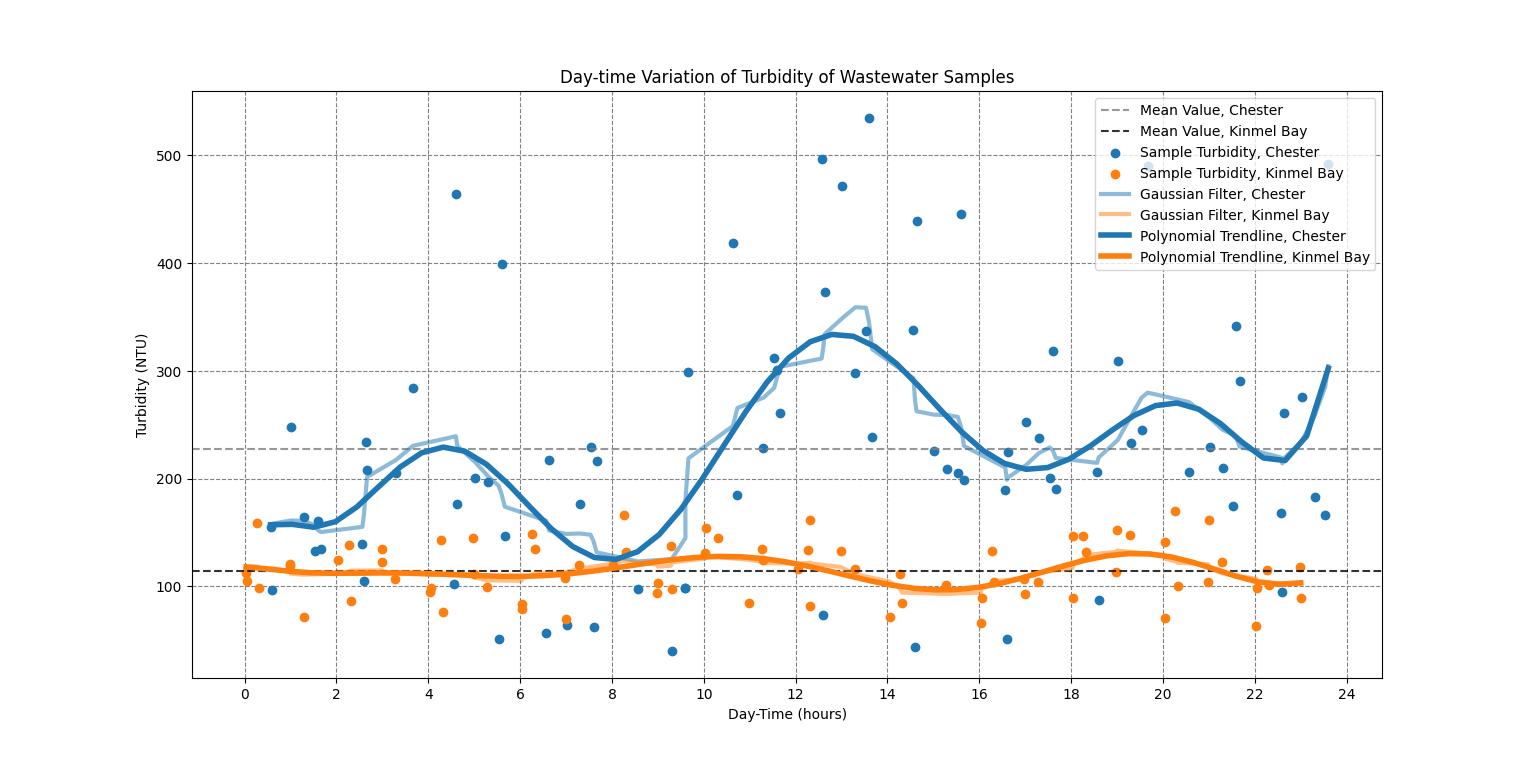


# Figure S2. Diurnal Variation of Wastewater Samples Turbidity at the Chester and Kinmel Bay WWTPs.

The polynomial function and a Gaussian function filter, sigma = 2, were applied to observe the trend during the day. Turbidity is highly variable for Chester samples but less variable for the Kinmel Bay samples. A peak in turbidity of wastewater samples from Chester occurred at 12:00 – 14:00 h.

**
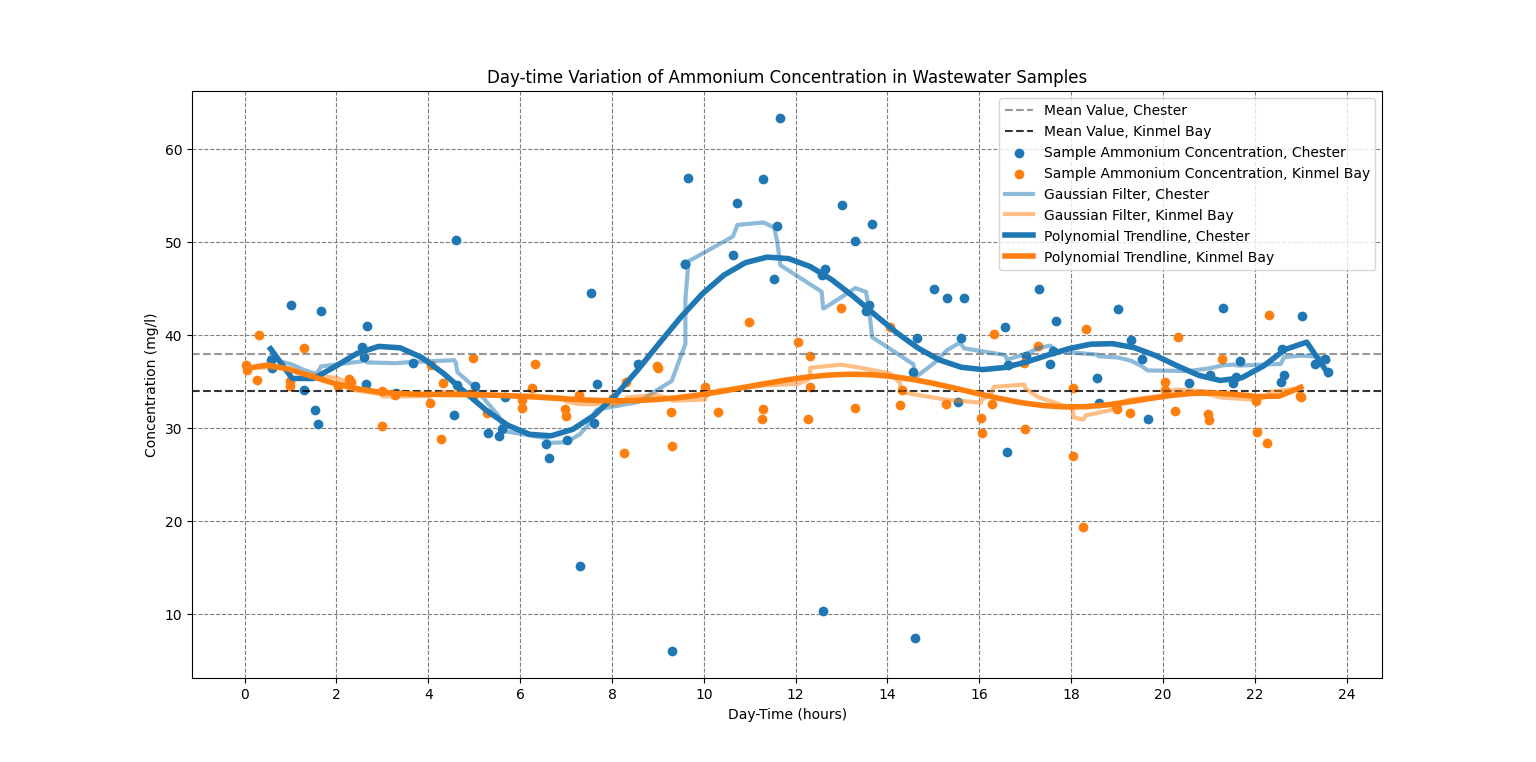
**

# Figure S3 Diurnal Variation of Ammonium Concentration in the Wastewater Samples Collected at Chester and Kinmel Bay WWTPs.

The polynomial function and a Gaussian function filter, sigma = 2, were applied to observe the trend during the day. The ammonium concentration is variable throughout the day for Chester wastewater samples and to a lesser extent for Kinmel Bay samples. For Chester, a significant concentration increase starts at 6:30, peaking at 11:30 h, and followed by a decrease from 12:00 to 16:00 h, after which the concentration remains relatively stable. Kinmel Bay trendlines indicate a small increase at 12:00 – 14:00 h. However, if the Kinmel Bay data-points are examined independently, then it can be observed that relatively there are more datapoints with concentrations >40 mg/l between 11:00 – 23:30 h.

**
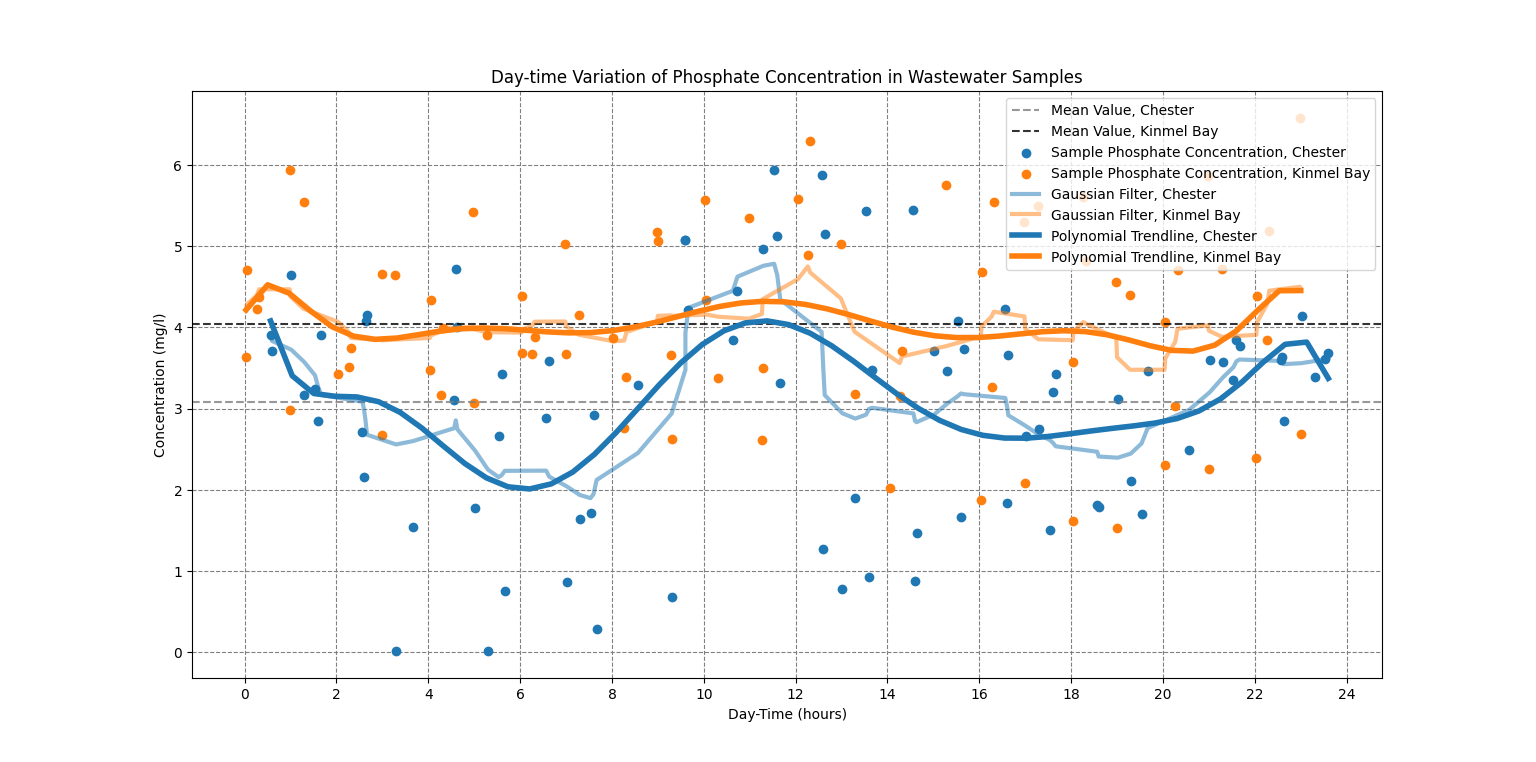
**

# Figure S4. Diurnal Variation of Phosphate Concentration in the Wastewater Samples Collected at Chester and Kinmel Bay WWTPs.

The polynomial function and a Gaussian function filter, sigma = 2, were applied to observe the trend during the day. The phosphate concentration is more variable for Chester samples than for Kinmel Bay samples. There is a concomitant increase in the concentration between 10:00 and 12:30 h according to the Gaussian filter function.

**
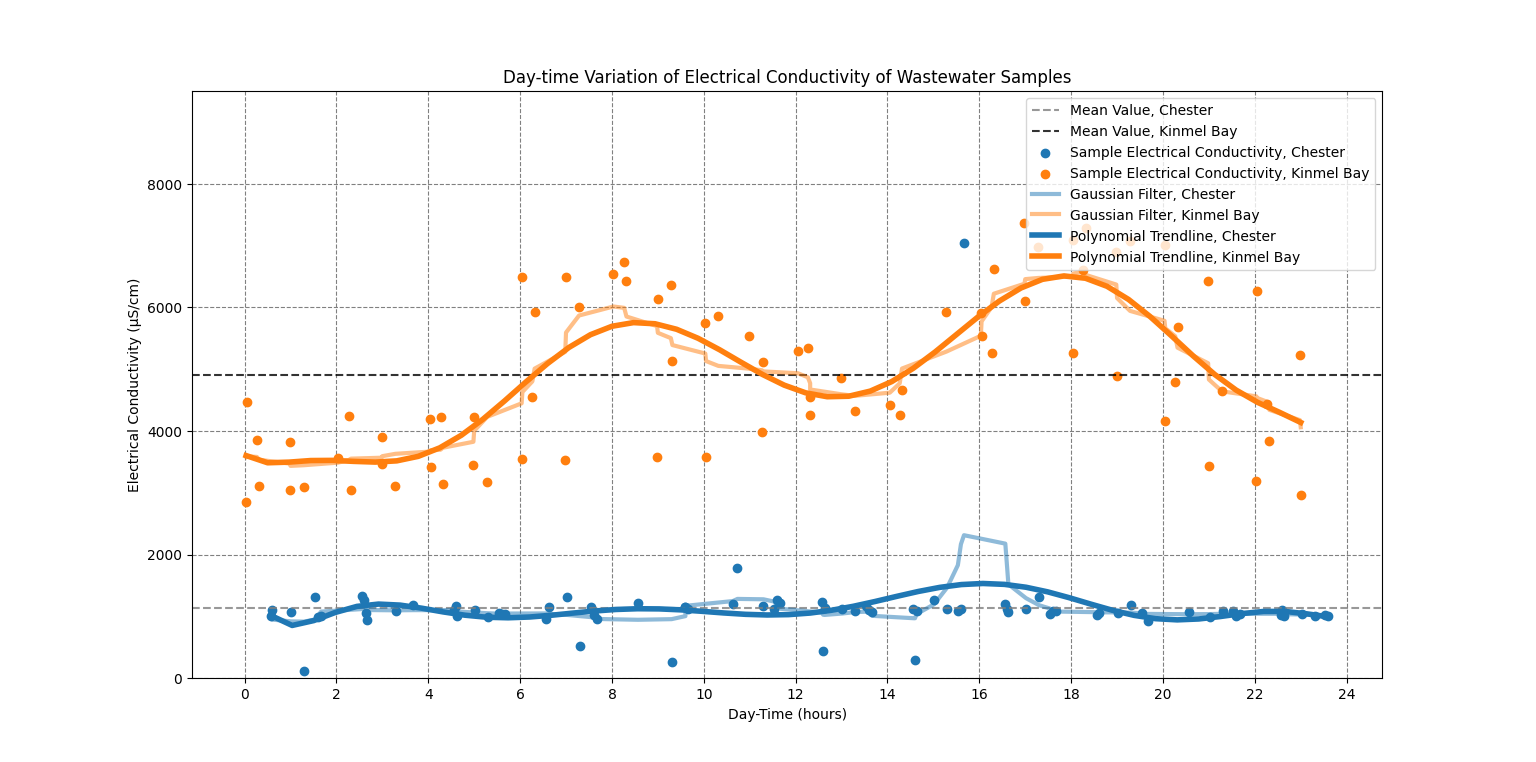
**

# **Figure S5. Diurnal Variation of Wastewater Sample Electrical conductivity at the Chester and Kinmel Bay WWTPs.**

The polynomial function and a Gaussian function filter, sigma = 2, were applied to observe the trend during the day. Electrical conductivity is more variable for Kinmel Bay than Chester samples. A noticeable increase happens for Kinmel Bay samples starting at 4:30, the peak being at 17:45, followed by a decrease from 18:30 to 24:00. The electrical conductivity of Chester samples is relatively stable throughout the daytime except for a peak at 15:30 – 16:30.


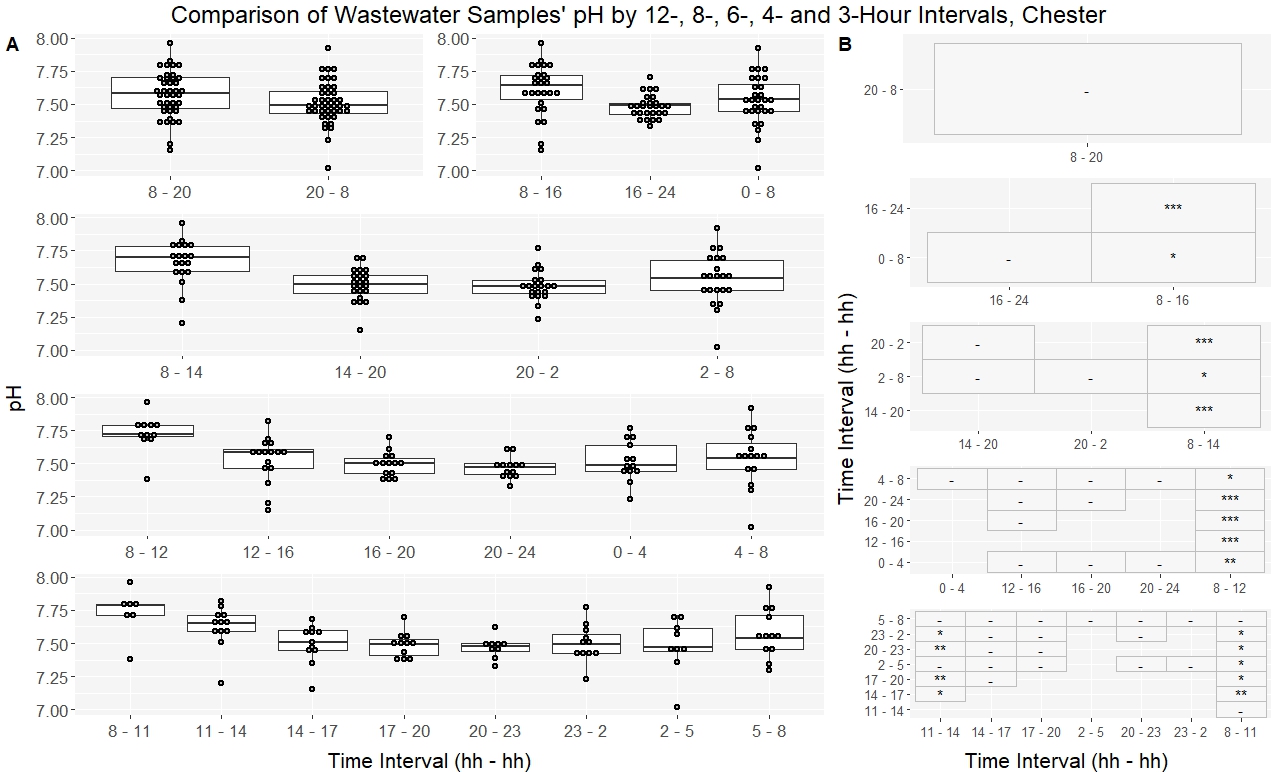


# Figure S6. Comparison of wastewater sample pH by 12-, 8-, 6-, 4- and 3-hour intervals at Chester


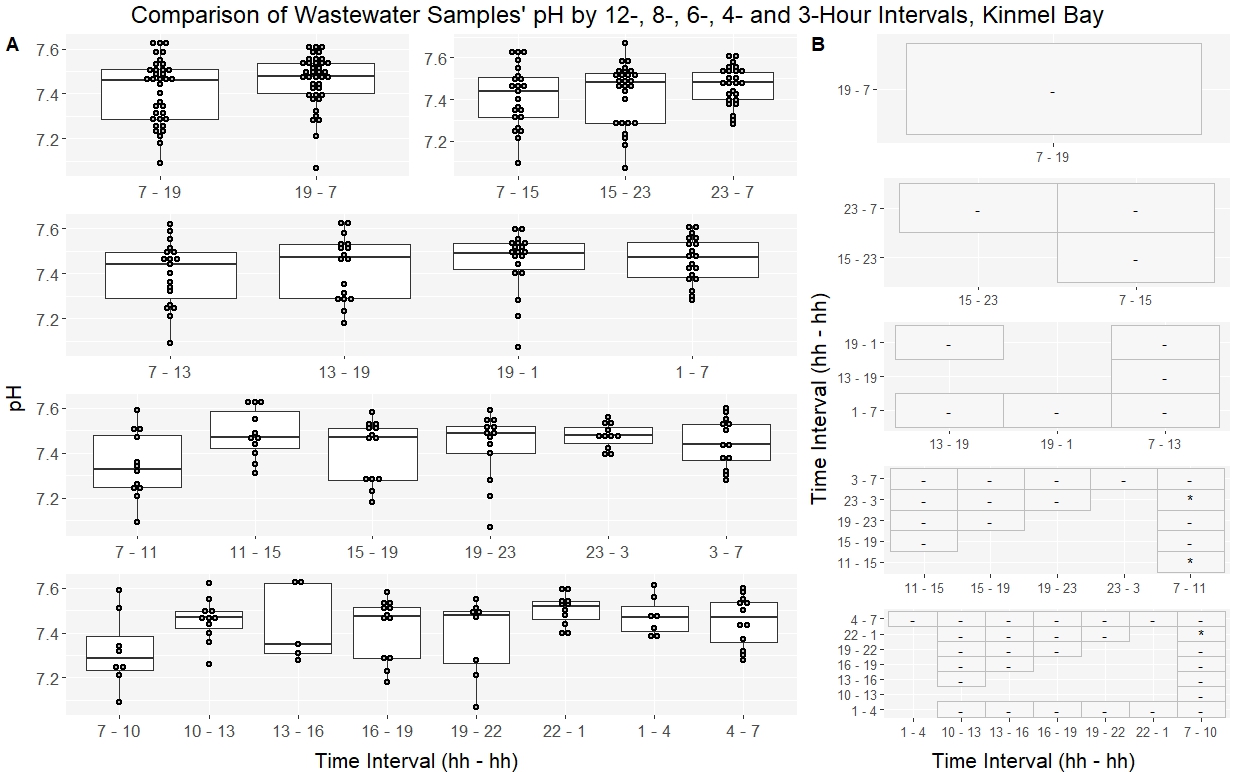


# Figure S7. Comparison of wastewater sample pH by 12-, 8-, 6-, 4- and 3-hour intervals at Kinmel Bay


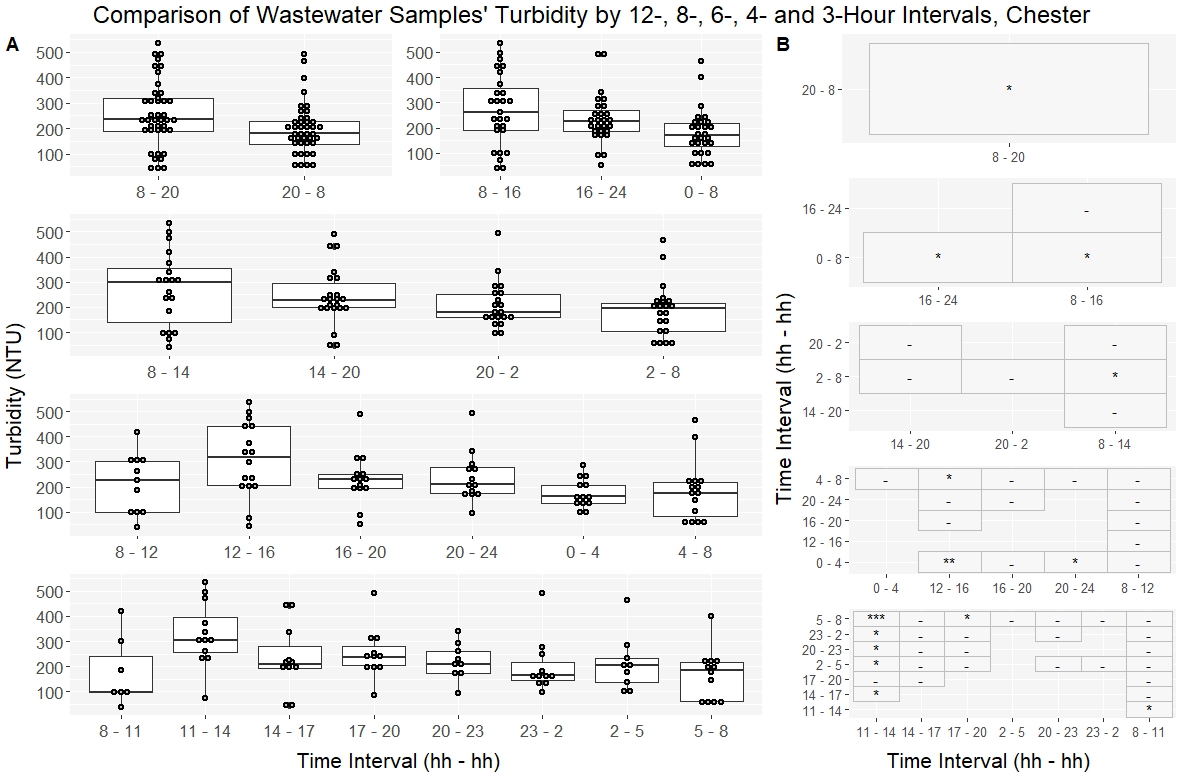


# Figure S8 Comparison of wastewater sample turbidity by 12-, 8-, 6-, 4- and 3-hour intervals at Chester


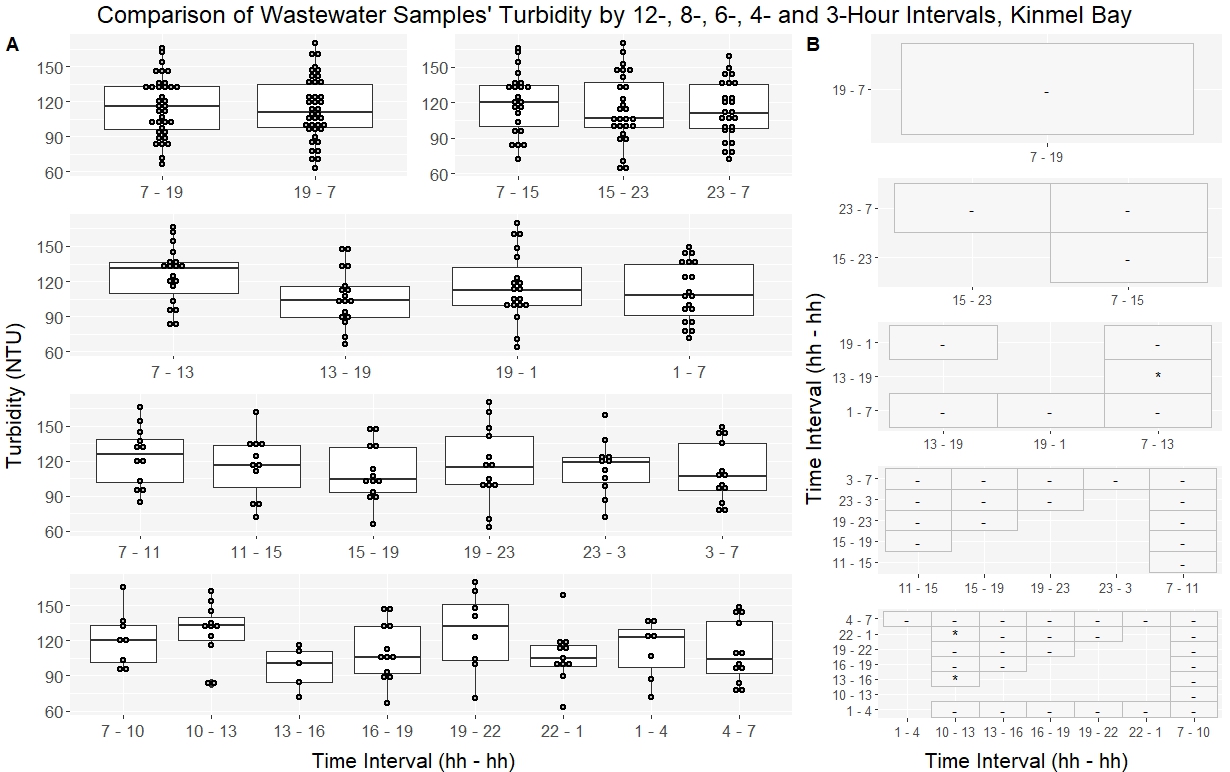
**Figure S9. Comparison of wastewater sample turbidity by 12-, 8-, 6-, 4- and 3-hour intervals at Kinmel Bay**


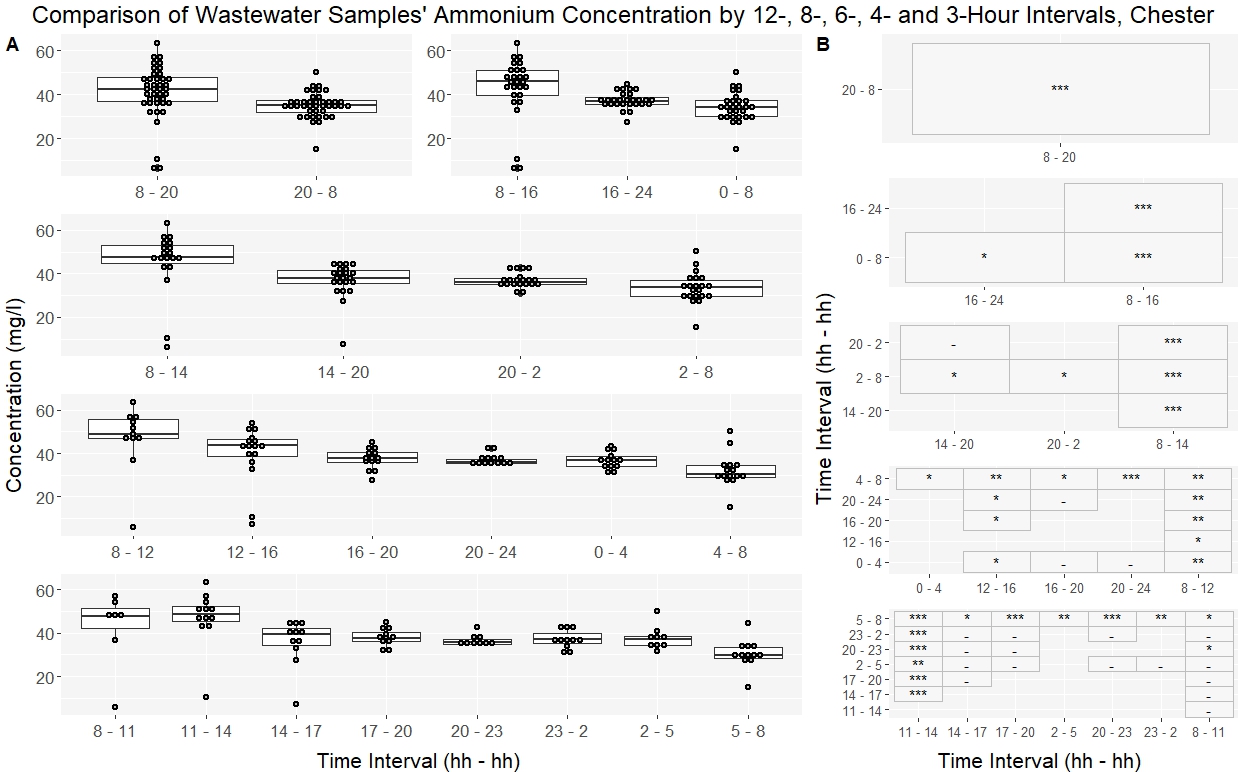


# Figure S10. Comparison of wastewater sample ammonium concentration by 12-, 8-, 6-, 4- and 3-hour intervals at Chester


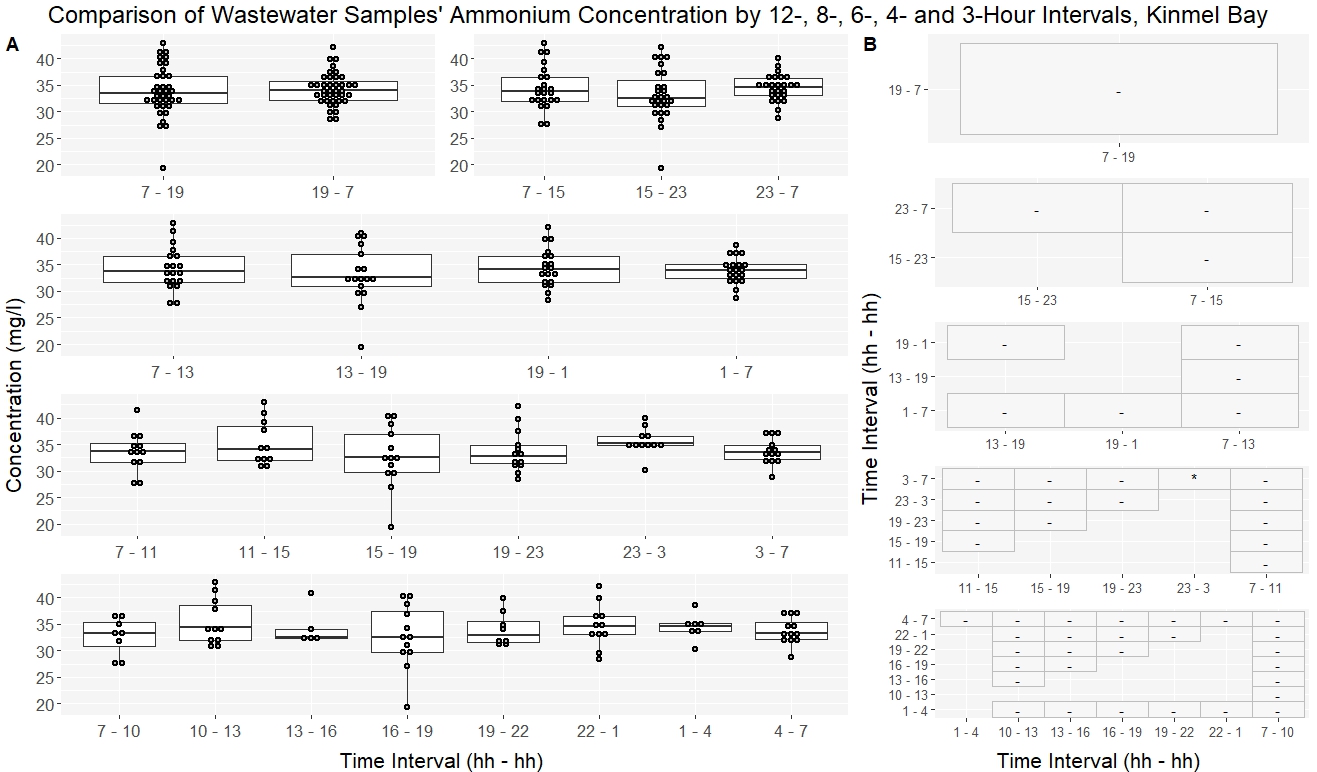


# Figure S11. Comparison of wastewater sample ammonium concentration by 12-, 8-, 6-, 4- and 3-hour intervals at Kinmel Bay


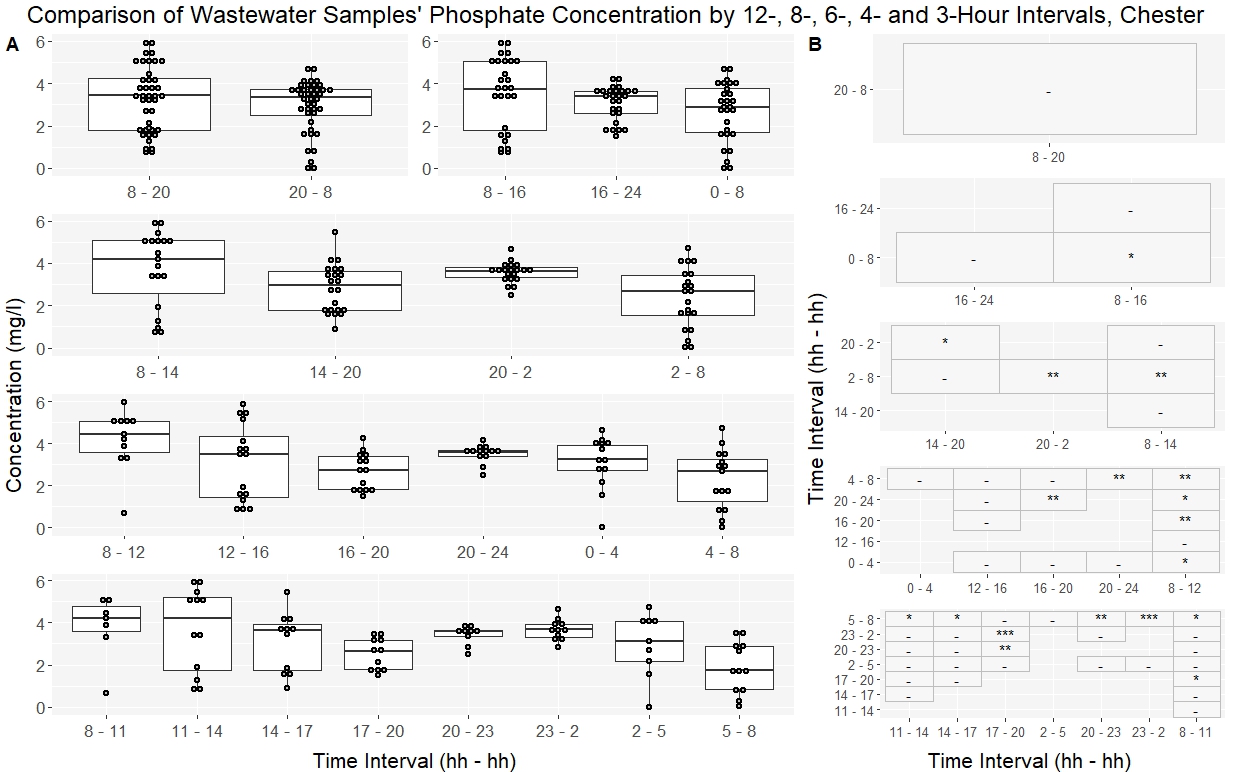


# Figure S12. Comparison of wastewater sample phosphate concentration by 12-, 8-, 6-, 4- and 3-hour intervals at Chester


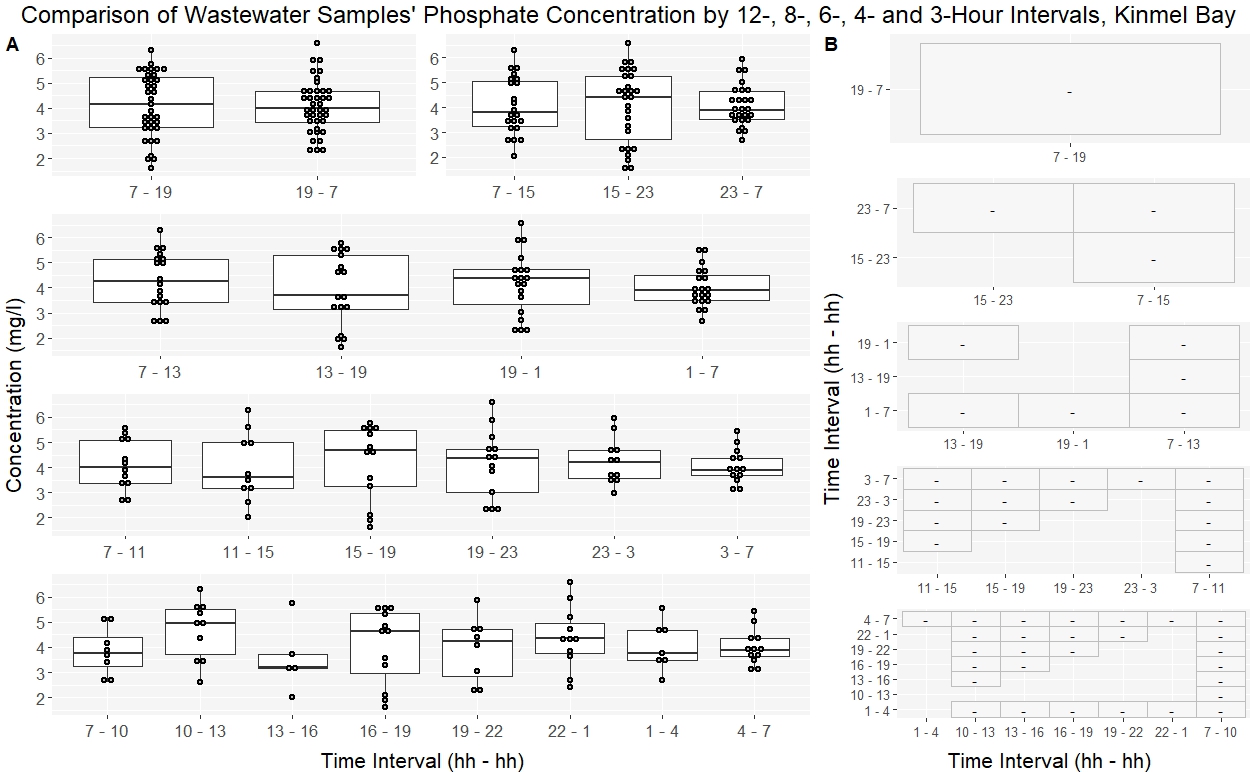
**Figure S13. Comparison of wastewater sample phosphate concentration by 12-, 8-, 6-, 4- and 3-hour intervals at Kinmel Bay**


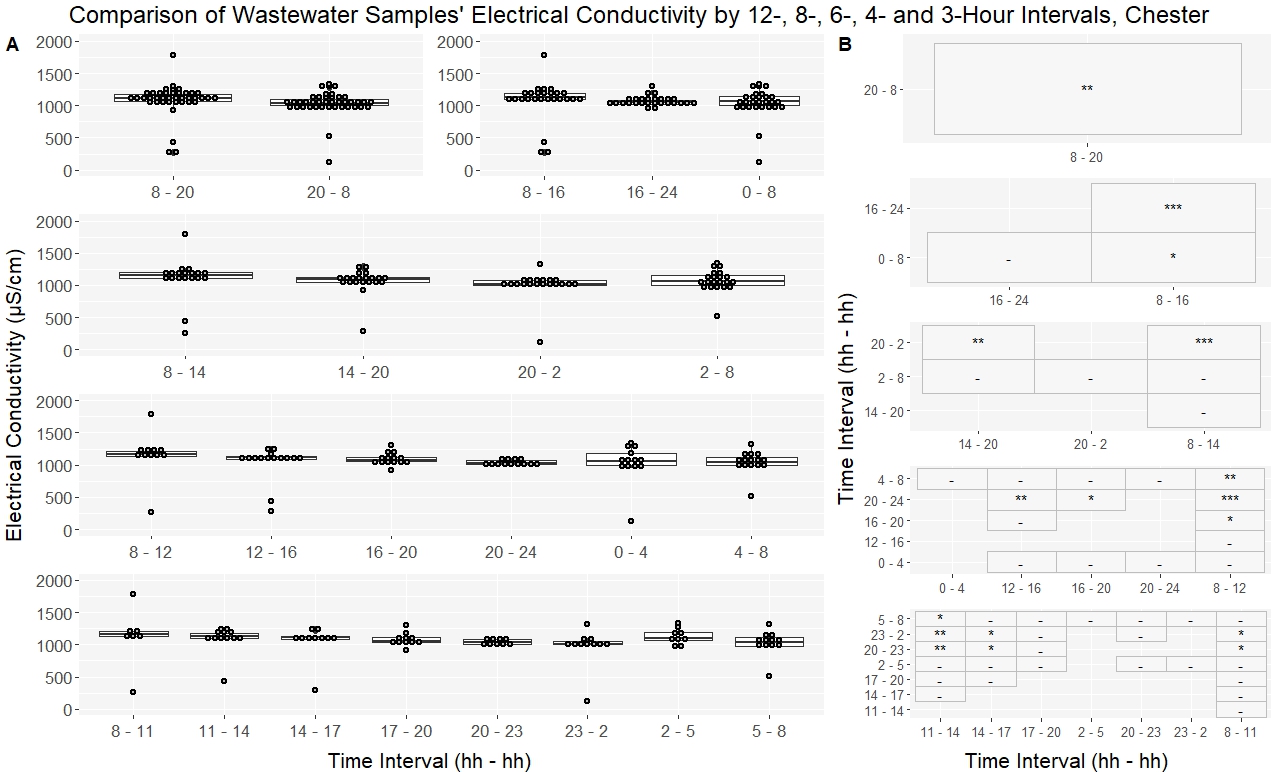


# Figure S14. Comparison of wastewater sample electrical conductivity by 12-, 8-, 6-, 4- and 3-hour intervals at Chester


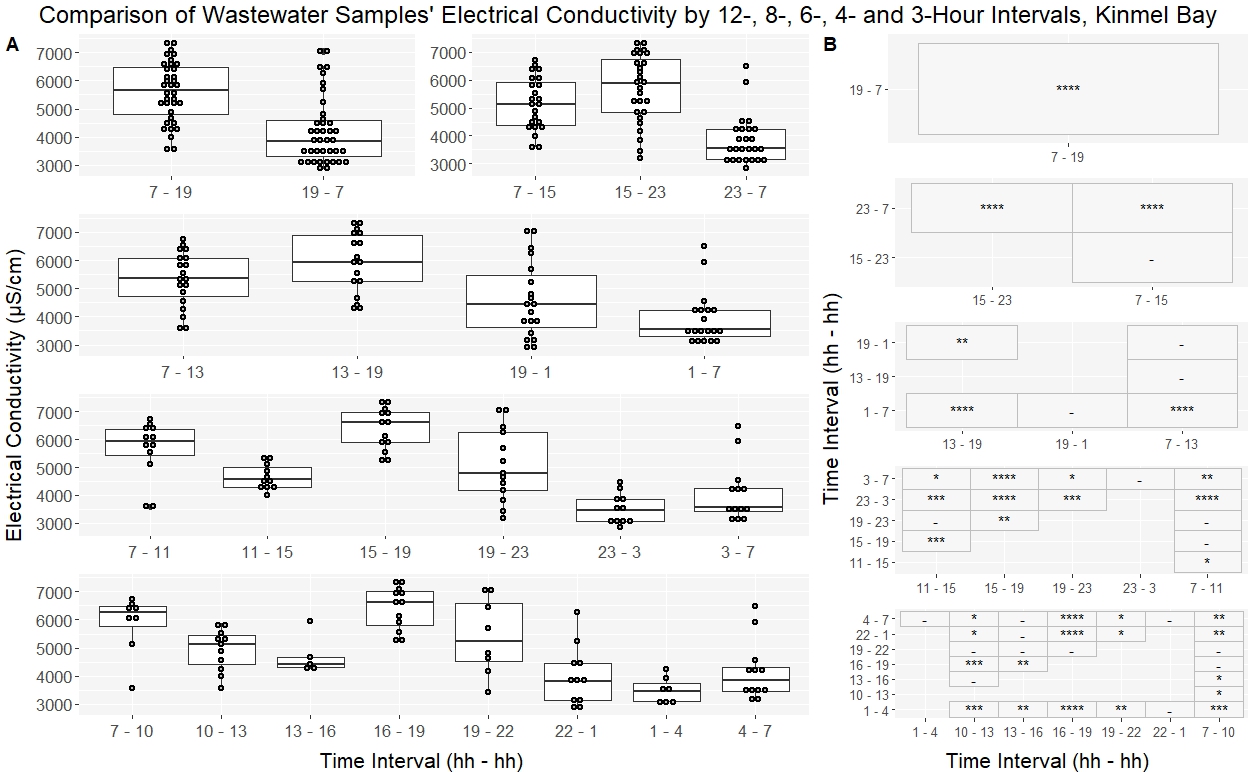


# Figure S15. Comparison of wastewater sample electrical conductivity by 12-, 8-, 6-, 4- and 3-hour intervals at Kinmel Bay


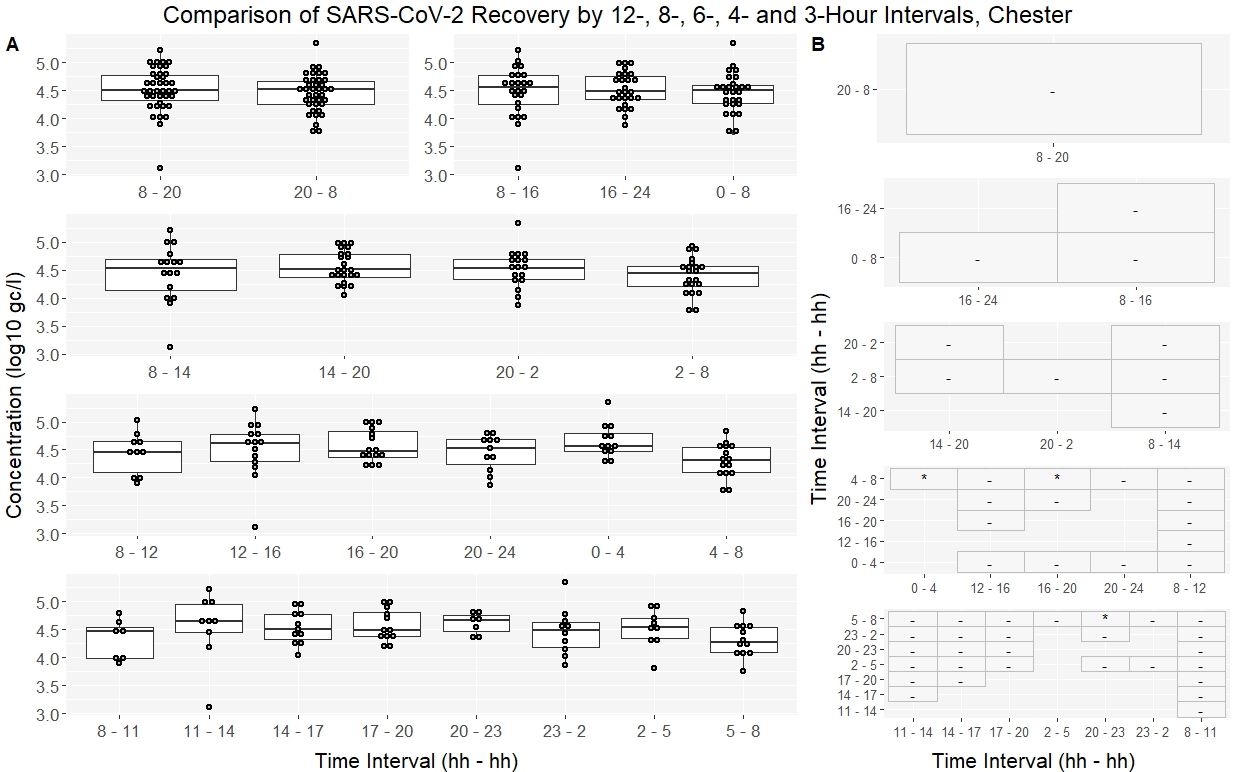


# Figure S16. Comparison of wastewater SARS-CoV-2 concentration by 12-, 8-, 6-, 4- and 3-hour intervals at Chester


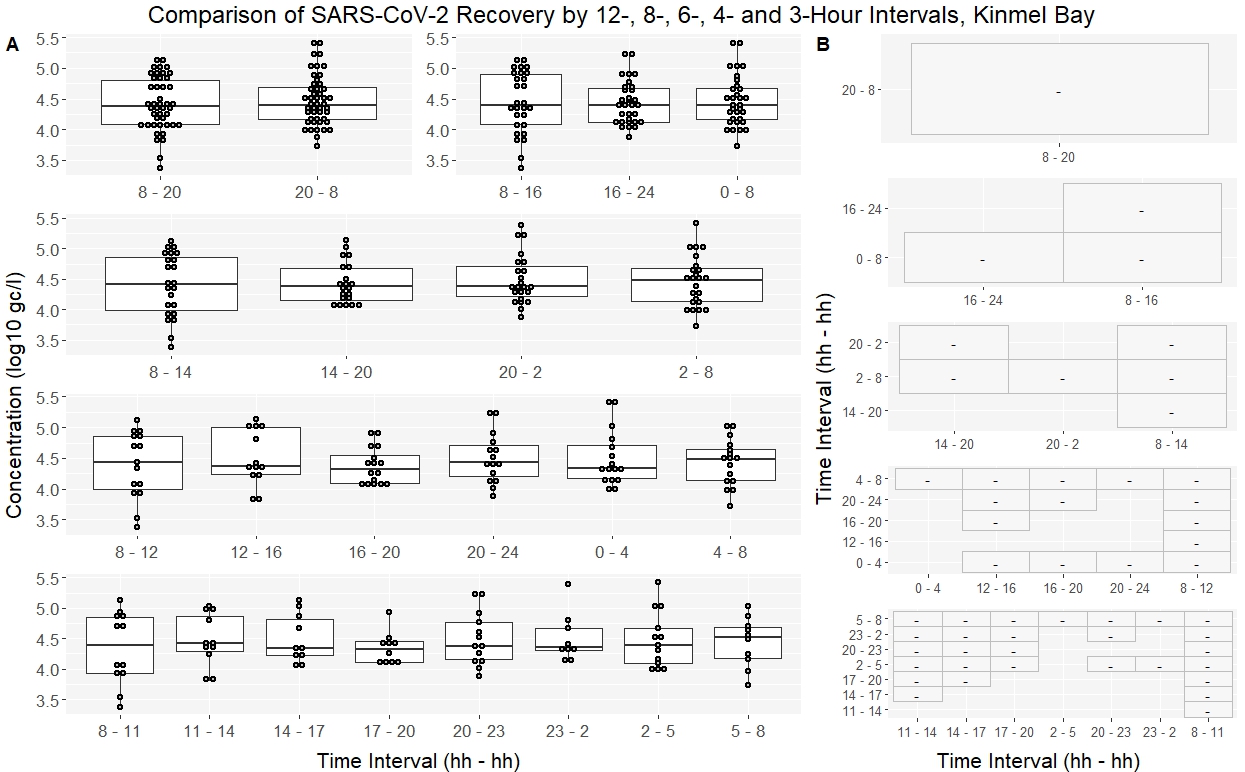


# Figure S17. Comparison of wastewater SARS-CoV-2 concentration by 12-, 8-, 6-, 4- and 3-hour intervals at Kinmel Bay


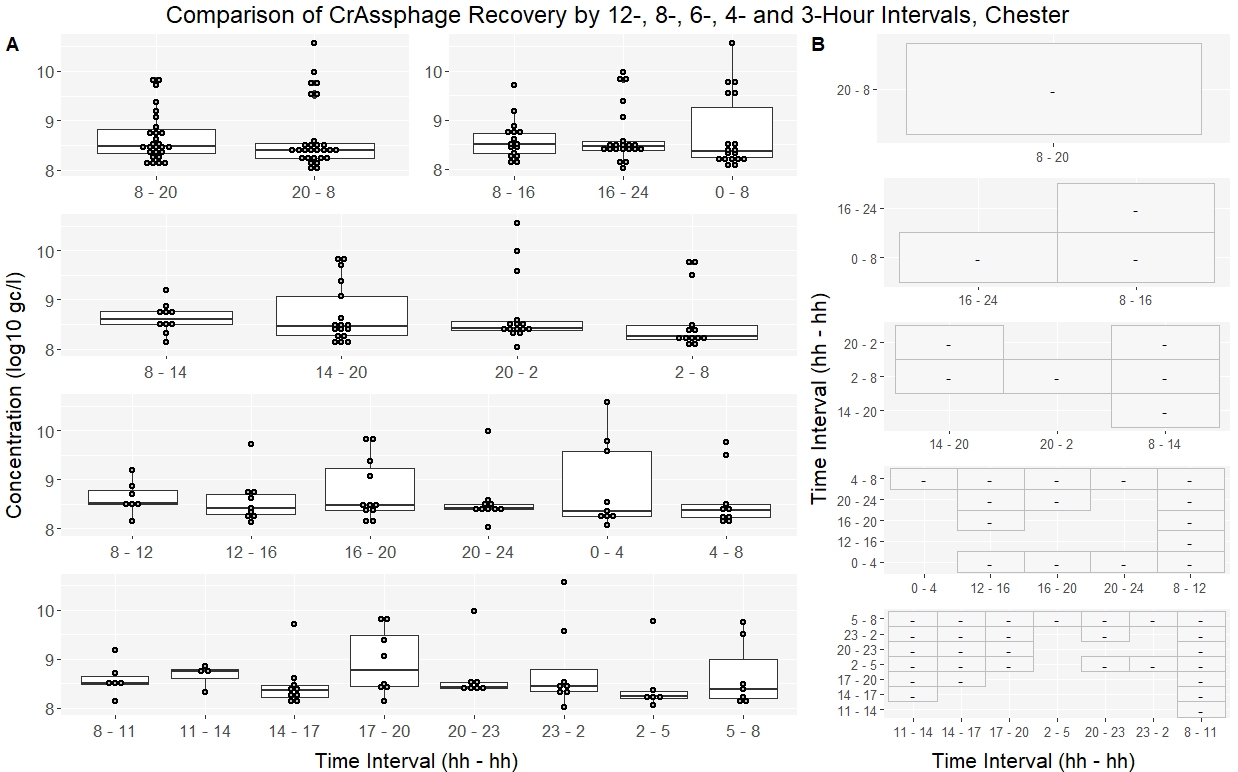


# Figure S18. Comparison of wastewater CrAssphage concentration by 12-, 8-, 6-, 4- and 3-hour intervals at Chester


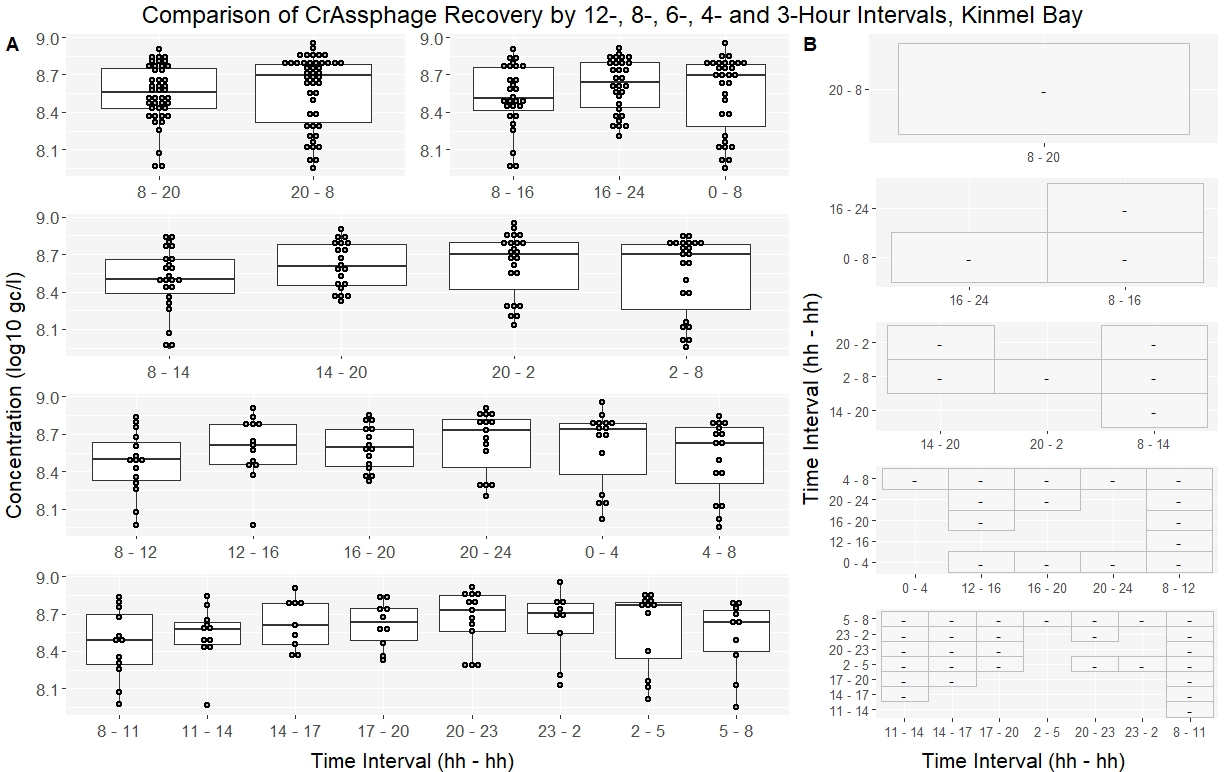


# Figure S19. Comparison of wastewater CrAssphage concentration by 12-, 8-, 6-, 4- and 3-hour intervals at Kinmel Bay

Table S1. Polynomial Trendline Characteristics of Various Wastewater Samples’ Parameters. The presented values for root mean square error (RMSE) and R^2^ values are for polynomial trendlines of 10th order as obtained previously.

| **Parameter Trendline** | **Chester** | | **Kinmel Bay** | |
| --- | --- | --- | --- | --- |
|  | **RMSE** | **R^2^** | **RMSE** | **R^2^** |
| SARS-CoV-2 Concentration | 4851.8 | 0.87509 | 5160.27 | 0.79238 |
| CrAssphage Concentration | 467880869 | 0.93722 | 27901186.5 | 0.7986 |
| pH | 0.026161 | 0.8989 | 0.013476 | 0.9268 |
| Electrical Conductivity | 197.22 | 0.43737 | 144.41279 | 0.97735 |
| Turbidity | 13.419678 | 0.9472 | 2.71445 | 0.912929 |
| Ammonium Concentration | 1.937349 | 0.85628 | 0.593797 | 0.79187 |
| Phosphate Concentration | 0.34486 | 0.74018 | 0.16405499 | 0.599465 |
